# Quaking isoforms cooperate to promote the mesenchymal phenotype

**DOI:** 10.1091/mbc.E23-08-0316

**Published:** 2024-01-12

**Authors:** Daniel P. Neumann, Caroline A. Phillips, Rachael Lumb, Helen M. Palethorpe, Yesha Ramani, Brett G. Hollier, Luke A. Selth, Cameron P. Bracken, Gregory J. Goodall, Philip A. Gregory

**Affiliations:** a Centre for Cancer Biology, University of South Australia and SA Pathology, Adelaide, South Australia 5000, Australia; b Australian Prostate Cancer Research Centre - Queensland, Centre for Genomics and Personalised Health, Faculty of Health, School of Biomedical Sciences, Queensland University of Technology, Translational Research Institute, Brisbane, Queensland 4102, Australia; c Flinders Health and Medical Research Institute and Freemasons Centre for Male Health and Wellbeing, Flinders University, Bedford Park, South Australia 5042, Australia; d Faculty of Health and Medical Sciences, and; e School of Biological Sciences, Faculty of Sciences, Engineering and Technology, The University of Adelaide, Adelaide, South Australia 5000, Australia; Emory University

## Abstract

The RNA-binding protein Quaking (QKI) has widespread effects on mRNA regulation including alternative splicing, stability, translation, and localization of target mRNAs. Recently, QKI was found to be induced during epithelial–mesenchymal transition (EMT), where it promotes a mesenchymal alternative splicing signature that contributes to the mesenchymal phenotype. QKI is itself alternatively spliced to produce three major isoforms, QKI-5, QKI-6, and QKI-7. While QKI-5 is primarily localized to the nucleus where it controls mesenchymal splicing during EMT, the functions of the two predominantly cytoplasmic isoforms, QKI-6 and QKI-7, in this context remain uncharacterized. Here we used CRISPR-mediated depletion of QKI in a human mammary epithelial cell model of EMT and studied the effects of expressing the QKI isoforms in isolation and in combination. QKI-5 was required to induce mesenchymal morphology, while combined expression of QKI-5 with either QKI-6 or QKI-7 further enhanced mesenchymal morphology and cell migration. In addition, we found that QKI-6 and QKI-7 can partially localize to the nucleus and contribute to alternative splicing of QKI target genes. These findings indicate that the QKI isoforms function in a dynamic and cooperative manner to promote the mesenchymal phenotype.

## Abstract (executive summary)

Epithelial–mesenchymal transition (EMT) plays important roles in tumor progression. The RNA-binding protein QKI promotes EMT but the contribution of individual QKI isoforms to this process are not well understood.

We developed a model of CRISPR-mediated depletion of QKI with expression of individual QKI isoforms in isolation and in combination. Using this model, we demonstrate that QKI-5 is required to induce mesenchymal morphology, while combined expression of QKI-5 with QKI-6 or QKI-7 further enhanced mesenchymal morphology and cell migration.

These findings indicate that the QKI isoforms function in a dynamic and cooperative manner to promote the mesenchymal phenotype.

## INTRODUCTION

Epithelial–mesenchymal transition (EMT) allows epithelial-derived tumor cells to transition to a mesenchymal phenotype, a phenomenon that contributes to disease progression and metastasis (Yang *et al.*, 2020). The epithelial microRNA family, miR-200, is a strong negative regulator of EMT, through repression of the EMT-promoting transcription factors ZEB1 and ZEB2 (Bracken *et al.*, 2008; Burk *et al.*, 2008; Gregory *et al.*, 2008; Park *et al.*, 2008). Recently, it was discovered that the RNA-binding protein, QKI, is a target of the miR-200 family and during EMT the loss of miR-200 expression leads to increased QKI protein (Pillman *et al.*, 2018). QKI regulates splicing of many mRNAs during EMT, which contributes to morphological and phenotypic changes associated with increased cancer invasiveness (Pillman *et al.*, 2018).

The *QKI* gene is itself alternatively spliced to produce three major isoforms, QKI-5, QKI-6, and QKI-7, with mRNAs of ∼5, 6, and 7 kb, respectively (Ebersole *et al.*, 1996). The predominant isoform expressed after EMT, QKI-5, is localized in the nucleus and is thought to be responsible for regulation of alternative splicing (Pillman *et al.*, 2018). The cytoplasmic isoforms QKI-6 and QKI-7 are also induced during EMT (Pillman *et al.*, 2018) but their functions in this context remain largely unknown. Previous studies of the QKI isoforms have shown that, in addition to regulating alternative splicing, they can also influence mRNA stability (Larocque *et al.*, 2005; Doukhanine *et al.*, 2010; Cochrane *et al.*, 2017; Thangaraj *et al.*, 2017), localization (Larocque *et al.*, 2002) and translation (Saccomanno *et al.*, 1999; de Bruin *et al.*, 2016; Yamagishi *et al.*, 2016; Sakers *et al.*, 2021). However, characterizing the individual functions of QKI isoforms is complicated by cross-isoform autoregulation (Fagg *et al.*, 2017), making it challenging to discern definitive conclusions about their functions.

To achieve a better understanding of how QKI isoforms function in EMT, we used CRISPR-Cas9 to generate mesenchymal human mammary epithelial (mesHMLE) cell lines with almost complete loss of endogenous QKI protein and, from these, generated QKI isoform–specific inducible cell lines. Depletion of QKI protein in mesHMLE cells reduced their migration, decreased mesenchymal morphology, and altered splicing of mesenchymal splicing events. By reconstituting each QKI isoform in isolation, we found that QKI-5 plays a major role in promoting mesenchymal morphology while QKI-6 and QKI-7 further enhance this morphology and the migratory capacity of cells. QKI-6 and QKI-7 could also partially localize to the nucleus and contribute to alternative splicing of QKI target genes. These findings indicate that the QKI isoforms function in a cooperative manner to enhance the mesenchymal phenotype.

## RESULTS

### QKI self-regulation alters the balance of QKI isoforms in favor of QKI-6 and QKI-7 as QKI levels increase

**FIGURE 1:**
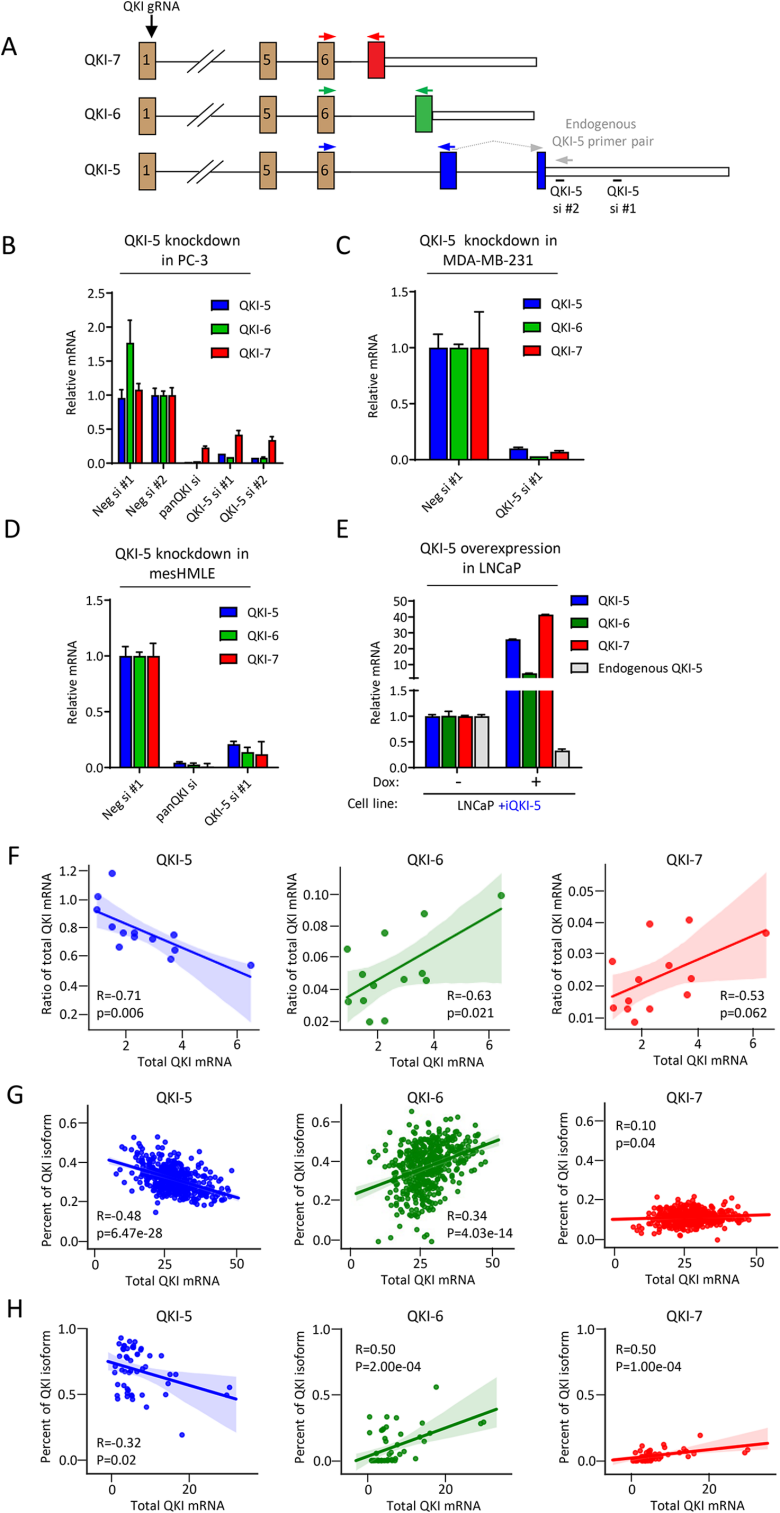
A relationship between the balance of the QKI isoforms and total QKI expression across tissues. (A) Schematic of the QKI locus including positions of the QKI gRNA, QKI-5 siRNAs, QKI isoform specific primers (colored along with isoform specific exon) and endogenous QKI-5 specific primer pair. (B–D) Quantitation of QKI isoforms following transfection of PC-3, MDA-MB-231, or mesHMLE cells with nontargeting (Neg si #1 and #2), panQKI targeting (siQKIpan), or QKI-5 specific (siQKI-5 #1, siQKI-5 #2) siRNAs. (E) Quantitation of QKI isoforms in untreated or 1.00 μg/ml doxycycline treated LNCaP-iQKI5 cells. (F) Ratios of QKI-5, QKI-6, and QKI-7 to total QKI mRNA plotted against total QKI mRNA of qRT-PCRs performed on RNA samples harvested from breast cancer cell lines. Expression of QKI-5, QKI-6, or QKI-7 expressed as a percentage of total QKI mRNA plotted against total QKI mRNA across (G) breast tissue (GTEx project) and (H) breast cancer cell line (CCLE) samples.

It has been previously reported that QKI engages in self-regulation including self-splicing in oligodendrocytes and myoblasts (Darbelli *et al.*, 2017; Fagg *et al.*, 2017). To determine the extent of QKI autoregulation in the context of breast and prostate cancer EMT, we examined the effect of knockdown of QKI-5 in a mesenchymal prostate cancer cell line (PC-3), a mesenchymal breast cancer cell line (MDA-MB-231), and a mesenchymal breast cell line (mesHMLE). Using small interfering RNA (siRNA) designed to target sequences specific to the QKI-5 mRNA (Figure 1A), we found that loss of QKI-5 also caused loss of QKI-6 and QKI-7 mRNA, consistent with the previous reports of QKI-5 controlling their alternative splicing (Figure 1, B–D).

We next overexpressed QKI-5 in an epithelial prostate cancer cell line (LNCaP) to confirm that increased QKI-5 expression is sufficient to upregulate QKI-6 and QKI-7 in this context. Consistent with the effect of QKI-5 knockdown, overexpression of QKI-5 caused an increase in QKI-6 and QKI-7 mRNA in both contexts (Figure 1E). To evaluate whether the increased production of QKI-6 and QKI-7 is accompanied by a decrease in spliced product of QKI-5, we designed a qRT-PCR primer pair that specifically amplifies endogenous and not the overexpressed QKI-5 (Figure 1A). Overexpression of QKI-5 in LNCaP cells decreased the amount of detectable endogenous QKI-5 mRNA, consistent with QKI-5 repressing its own transcript via alternative splicing to generate QKI-6 and QKI-7 mRNAs (Figure 1E).

We reasoned that if QKI-5 represses its own transcript in favor of QKI-6 and QKI-7, then in contexts where QKI is more highly expressed, QKI-6 and QKI-7 would constitute a higher proportion of the total pool of QKI mRNA. To assess this, we performed qRT-PCRs for each QKI isoform and total QKI mRNA on a panel of breast cancer cell lines and plotted the ratio of each isoform to total QKI against total QKI expression. Indeed, the ratio of QKI-5 to total QKI was negatively correlated, and the ratio of QKI-6 and QKI-7 to total QKI was positively correlated (Figure 1F, relative mRNA expression plotted in Supplemental Figure 1A). To determine whether this observation is broadly consistent in other contexts, we conducted a survey of QKI isoform expression across the Genotype-Tissue Expression (GTEx) and the Cancer Cell line Encyclopedia (CCLE) datasets (Barretina *et al.*, 2012; Consortium, 2013). In both breast tissue and breast cancer cell lines, we found the ratio of QKI-6 and QKI-7 to total QKI was higher in samples with greater total QKI mRNA levels (Figure 1, G and H). This relationship was also observed across different tissue types in both the CCLE and GTEx project, where tissues and cell lines derived from neuronal sources, which tend to express higher total QKI, expressed a higher ratio of QKI-6 and QKI-7 to total QKI than samples from other tissue types (Supplemental Figure 1, B–G). Collectively, these findings suggest that QKI-self splicing affects the balance of the QKI isoforms across tissues.

### The generation of mesenchymal cell lines expressing single QKI isoforms

**FIGURE 2:**
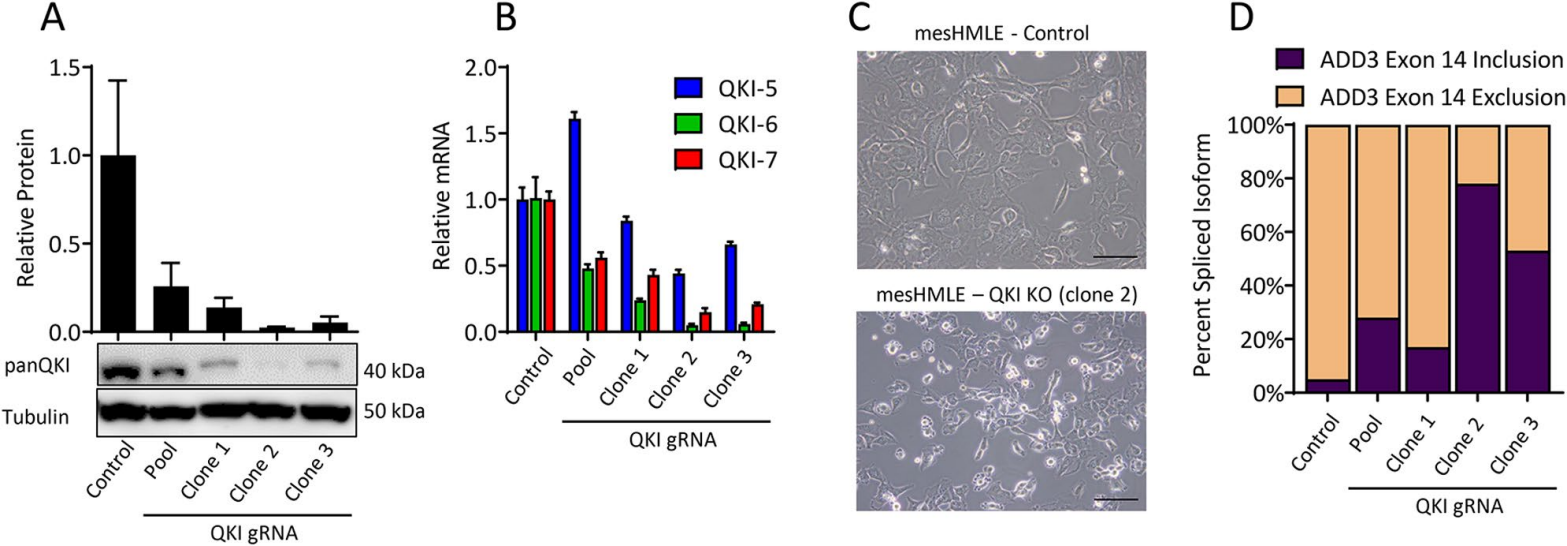
Loss of QKI evokes an epithelial-like morphology in mesenchymal breast cells. (A) Western blot for panQKI in mesHMLE control cells and mesHMLEs transfected with a QKI gRNA (pool and resulting single cell clones). This western blot was performed three times and the bar chart represents the mean –/+ SD. (B and D) Quantitative RT-PCR for the QKI isoforms and ADD3 isoforms. For D, the bar chart shows relative percentage of the ADD3 transcripts with exon 14 included or excluded. Data for qRT-PCRs is represented as the mean of three technical replicates ± SD. (C) Phase images of mesHMLE wild-type (control) and QKI KO (clone #2) cells. Scale bars represent 50 µm.

The nuclear localized QKI isoform, QKI-5, has previously been shown to influence mesenchymal features of breast cancer cells, including promoting an elongated cell morphology, increasing cell migration, and facilitating a mesenchymal alternative splicing program (Li *et al.*, 2018; Pillman *et al.*, 2018). While the levels of all QKI isoforms increase during EMT and are higher in mesenchymal compared with epithelial breast cancer cells (Pillman *et al.*, 2018), it is unclear whether QKI-6 and -7 play a role in the mesenchymal state. Autoregulation between the QKI isoforms, including self-splicing and influences on each other's mRNA levels and translation (Fagg *et al.*, 2017), makes it difficult to determine the contribution of each isoform to the mesenchymal phenotype. To overcome these effects, we used CRISPR-Cas9 to strongly deplete endogenous QKI levels and ectopically expressed individual QKI isoforms in isolation to allow the study of their individual functions. Following transient transfection of mesHMLE cells with a CRISPR-Cas9 complex containing a gRNA targeted to exon 1 of QKI shared across all QKI isoforms (Figure 1A), single cell-derived clones were screened by Western blot for loss of QKI protein. Several single-cell clones were isolated, and we identified a clone with reduced QKI isoform mRNA levels and a strong reduction of total QKI protein (∼40-fold) by Western blot (Clone 2, Figure 2, A and B), hereafter referred to as mesHMLE-QKI-KO cells.

Similar to our previous study of transient loss of QKI in mesenchymal breast cell lines (Pillman *et al.*, 2018), we found the mesHMLE-QKI-KO cells to be smaller and less elongated than wild-type cells (Figure 2C). Furthermore, we found a strong switch to the exon 14-retaining epithelial isoform of ADD3, a key alternative splice event directly regulated by QKI-5 (Yang *et al.*, 2016; Pillman *et al.*, 2018; Wang *et al.*, 2021; Figure 2D). To characterize the genomic alterations that were responsible for the disruption of QKI protein production, we performed Sanger sequencing (Supplemental Figure 2). This revealed three QKI alleles present within mesHMLE-QKI-KO cells, two of which (allele one and three) contained mutations that cause a frameshift and the other (allele 2) containing a three-nucleotide deletion that could still generate a protein product and likely accounts for the small amount of QKI protein that is still detectable (Supplemental Figure 2). We reasoned that the strong changes in QKI protein levels, morphology, and alternative splicing observed in this clone indicated that the remaining allele had little functional consequence and that mesHMLE-QKI-KO cells harbored a significant disruption of QKI function.

To reconstitute the expression of the QKI isoforms, the QKI-5, QKI-6, and QKI-7 open reading frames (ORF) were cloned into the doxycycline-inducible expression vector pInducer20 (Meerbrey *et al.*, 2011) (Supplemental Figure 3). This led to the generation of mesHMLE-QKI-KO-iQKI-5, mesHMLE-QKI-KO-iQKI-6, and mesHMLE-QKI-KO-iQKI-7 cell lines. To enable coexpression of two isoforms simultaneously, we cloned the QKI-5 ORF into the pLX-301 constitutive expression vector (Supplemental Figure 3) and transduced mesHMLE-QKI-KO cells with this vector along with either pInducer20-QKI-6 or pInducer20-QKI-7, leading to the generation of mesHMLE-QKI-KO-cQKI-5-iQKI-6 and mesHMLE-QKI-KO-cQKI-5-iQKI-7 cell lines.

### QKI isoforms each contribute to QKI-mediated alternative splicing in mesenchymal cells

QKI-5 is assumed to be the driver of QKI-mediated functions in alternative splicing as it is the only isoform with a nuclear localization signal. However, the Qua1 domain is common to all isoforms, and allows them to dimerize with each other (Pilotte *et al.*, 2001), suggesting that the cytoplasmic isoforms could potentially shuttle into the nucleus as a heterodimer with QKI-5 and participate in splicing regulation.

**FIGURE 3:**
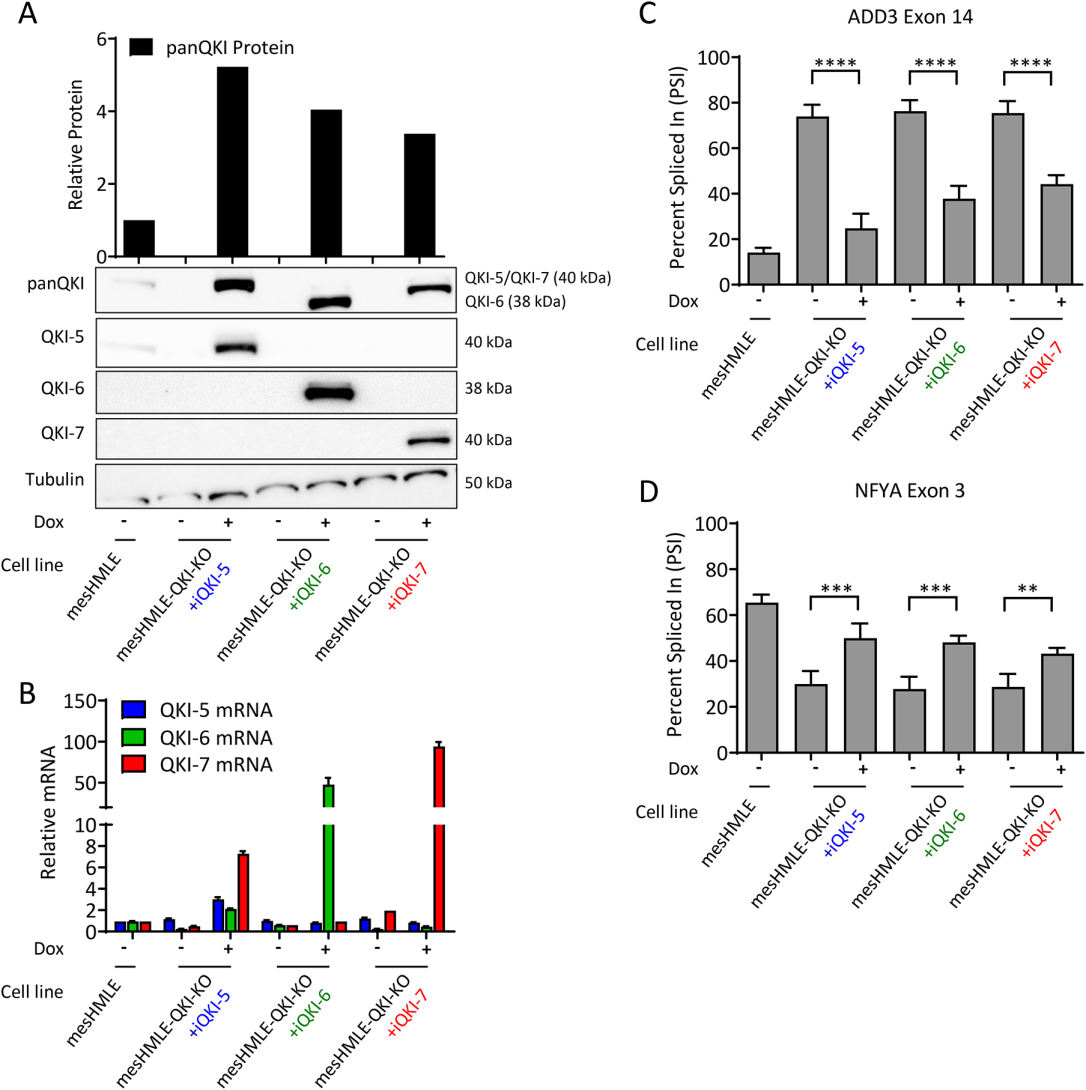
All QKI isoforms can participate in alternative splicing. (A) Western blot of total QKI and individual QKI isoform protein following 1.00 μg/ml doxycycline induction of each isoform in QKI knockout mesHMLE cells. The panQKI western blot is quantitated in the upper panel. (B) Quantitative RT-PCR of QKI isoform mRNA levels in the same samples as (A). Data is represented as the mean of three technical replicates ± SD. Quantitation by qRT-PCR of percent spliced in of (C) ADD3 exon 14 and (D) NFYA exon 3 in the same samples as (A) from three biological replicates ± SD. *P* values are calculated using one-way ANOVA (with Bonferroni multiple testing) and indicated as **p < 0.01, ***p < 0.001, and ****P < 0.0001.

To determine whether QKI-5, QKI-6, or QKI-7 can rescue the alterations in splicing caused by loss of endogenous QKI-5 protein, we examined the expression of two key QKI spliced targets, ADD3 (exon 14 exclusion) and NFYA (exon 3 inclusion; Pillman *et al.*, 2018), following doxycycline induction of each QKI isoform. In all three mesHMLE-QKI-KO–derived cell lines, total QKI protein was induced to similar levels with each specific isoform being induced at the RNA and protein level (Figure 3, A and B). Consistent with previous results (Figure 1E), QKI-5 overexpression also increased production of QKI-6 and QKI-7 mRNA (Figure 3B). Induction of each QKI isoform was able to promote splicing of ADD3 and NFYA (Figure 3, C and D). However, none of the QKI isoforms were able to rescue levels of splicing to wild-type levels, despite being more highly expressed than QKI in wild-type cells (Figure 3A). The ability of QKI-6 and QKI-7 to promote splicing could arise indirectly through secondary effects, by direct localization of these isoforms into the nucleus, or potentially through their nuclear shuttling via dimerizing with a small amount of residual QKI-5. These data confirm that all three QKI isoforms can contribute to alternative splicing and suggest they cooperate to mediate this process.

### Partial nuclear localization of QKI-6 and QKI-7 correlates with enhanced mesenchymal alternative splicing

To determine whether the level of QKI isoform overexpression influences their alternative splicing activity and impacts their localization, the QKI-inducible cell lines were cultured with increasing concentrations of doxycycline and stained for the specific isoform that was induced. RNA from cells treated under identical conditions was harvested to examine expression of each QKI isoform mRNA and the splicing of NFYA and ADD3. At the mRNA level, QKI-5, QKI-6, and QKI-7 were each expressed in a dose-dependent manner, which largely correlated with dose-dependent changes in splicing of ADD3 and NFYA, consistent with each QKI isoform having the capacity to promote QKI-associated alternative splicing (Supplemental Figure 4).

**FIGURE 4:**
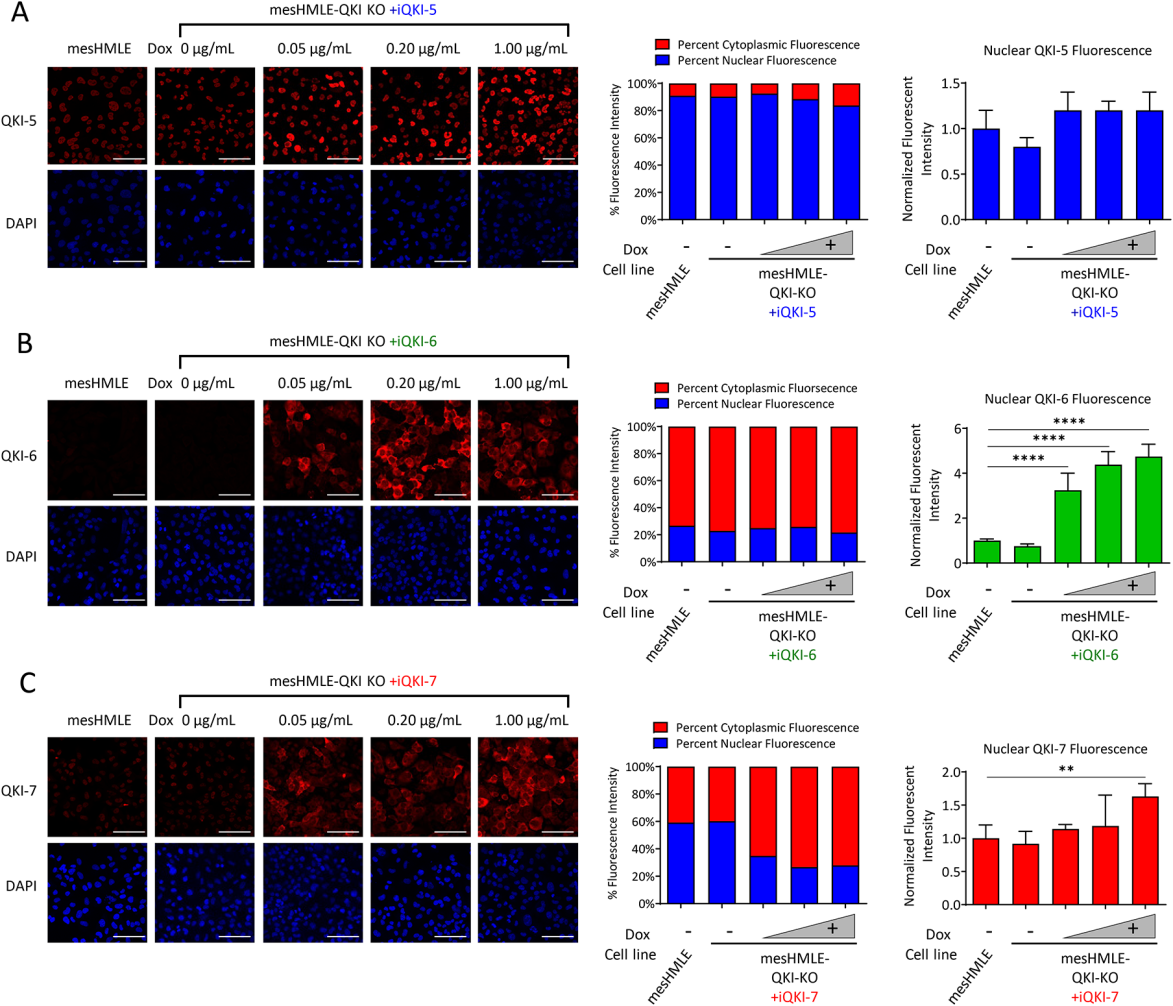
The cytoplasmic QKI isoforms can partially localize to the nucleus when overexpressed. Fluorescent microscopy images of overexpressed individual QKI isoforms in mesHMLE-QKI-KO-iQKI-5 (A), -QKI-6 (B) or iQKI-7 (C) cells cultured for 72 h without or with 0.05, 0.20, or 1.00 μg/ml doxycycline. Scale bars represent 50 µm. Quantitation of percentage nuclear and cytoplasmic isoform fluorescence per cell (middle panel) and total nuclear isoform fluorescence per cell for each cell line are plotted (right panel). All fluorescence is normalized to wild-type cells. Error bars represent ± SD of the mean of five separate images with cell numbers for iQKI-5 (300, 294, 301, 397, and 364), iQKI-6 (388, 370, 395, 307, and 296), and iQKI-7 (322, 414, 346, 395, and 432) experiments indicated in brackets (values are for mesHMLE, 0, 0.05, 0.20, and 1.00 μg/ml doxycycline, respectively). *P* values are calculated using one-way ANOVA (with Bonferroni multiple testing) and indicated as **p < 0.01 and ****p < 0.0001.

To determine the subcellular localization of the QKI isoforms, nuclear segmentation was performed using DAPI staining as a mask to calculate relative nuclear fluorescent intensity and percent nuclear to cytoplasmic intensity for each QKI isoform. There were distinct patterns of nuclear to cytoplasmic distribution for each isoform. In both wild-type mesHMLE and in untreated mesHMLE-QKI-KO-iQKI-5 cells, greater than 90% of QKI-5 is nuclear localized (Figure 4A). While treatment with 0.05 μg/ml doxycycline increase nuclear localization slightly, higher doses led to a higher percentage of QKI-5 being localized in the cytoplasm (Figure 4A), providing a potential explanation for why the effect on splicing plateaus at doses greater than this concentration (Supplementary Figure 4). Localization of QKI-6 was predominantly cytoplasmic and greater than 70% of all QKI-6 protein was localized in the cytoplasm (Figure 4B). However, nuclear QKI-6 fluorescence increased in a dose-dependent manner with increasing doxycycline concentration (Figure 4B). Interestingly, QKI-7 staining is mostly nuclear in wild-type and untreated mesHMLE-QKI-KO-iQKI-7 cells but becomes predominantly cytoplasmic when treated with higher levels of doxycycline (Figure 4C). Even though most of the increasing QKI-7 fluorescence is cytoplasmic, nuclear QKI-7 fluorescence also modestly increases with higher concentrations of doxycycline (Figure 4C). These data indicate that nuclear localization of QKI-6 and QKI-7 is enhanced when they are expressed at higher levels, consistent with their potential direct role in regulating the alternative splicing of ADD3 and NFYA.

### Nuclear localization of QKI-6 and QKI-7 is enhanced by QKI-5 overexpression

**FIGURE 5:**
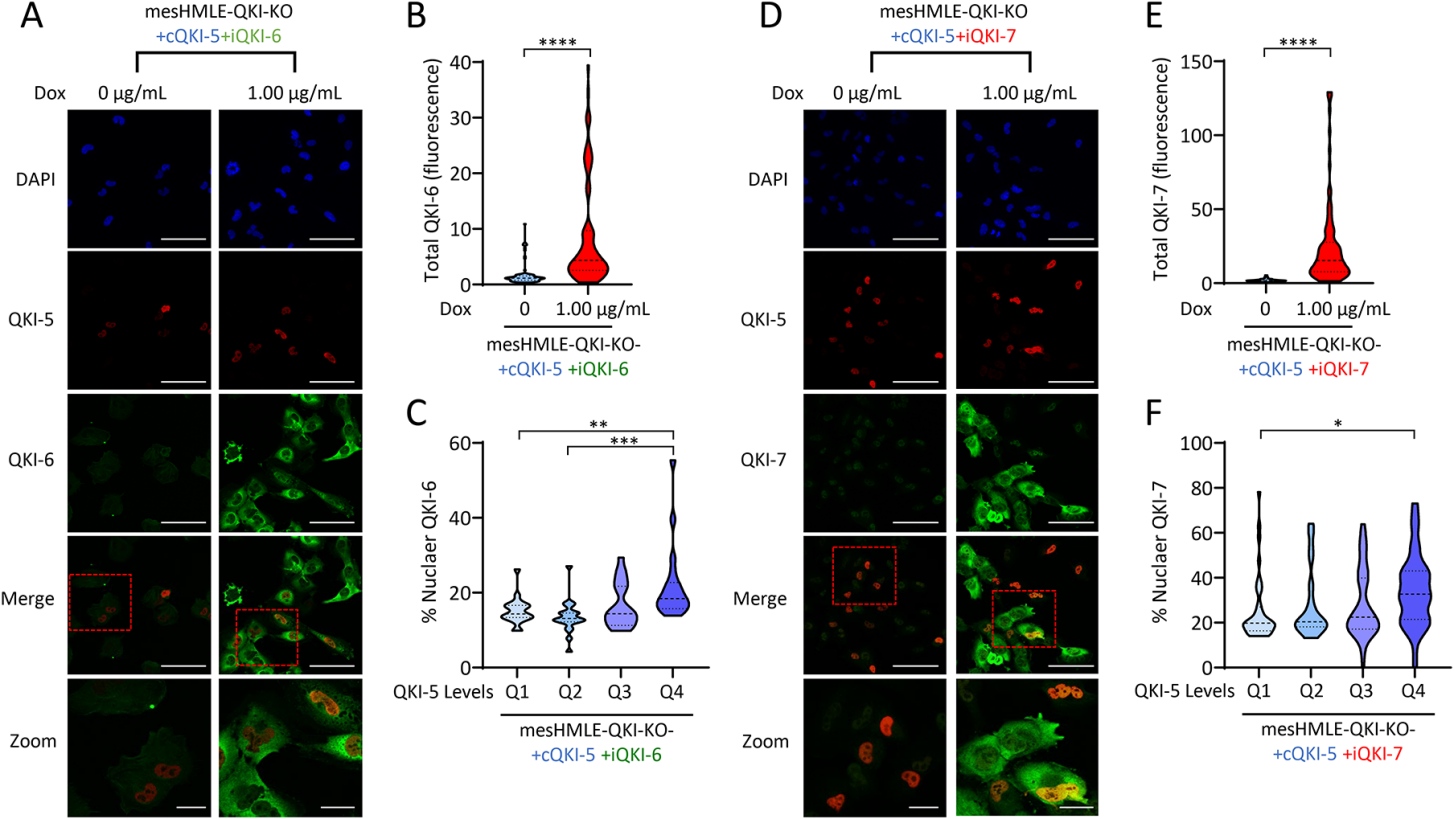
Nuclear localization of QKI-6 and -7 is enhanced by QKI-5 overexpression. Fluorescent microscopy images of the mesHMLE-QKI-KO-cQKI-5-iQKI-6 (A) and iQKI-7 (D) cell lines after culturing for 72 h with 1.0 µg/ml doxycycline. Cells were stained for QKI-5 (red) and either QKI-6 or QKI-7 (green), and counterstained with DAPI (blue). Merged images are the combination of QKI-5 and QKI-6 or QKI-7 alone. Large scale bars represent 50 µm and small scale bars represent 5 µm. Violin plots indicate total QKI-6 (B) or QKI-7 (E) fluorescence and percent nuclear QKI-6 (C) or QKI-7 (F) fluorescence with cell numbers for iQKI-6 (100, 77) and iQKI-7 (66, 91) experiments indicated in brackets (values are for 0 Dox and 1.00 μg/ml doxycycline, respectively). The interquartile ranges of QKI-5 fluorescent intensity are labeled from lowest to highest intensity as Q1, Q2, Q3, and Q4. *P* values are calculated using one-way ANOVA (with Bonferroni multiple testing) and indicated as *p < 0.05, **p < 0.01, *** *p* < 0.001, and ****p < 0.0001.

The observation that QKI-6 and QKI-7 can partially localize to the nucleus and participate in splicing could be explained by heterodimeric shuttling with residual QKI-5 in the mesHMLE-QKI-KO cell line. To examine the effect that QKI-5 expression has on QKI-6 and QKI-7 localization, we overexpressed constitutive QKI-5 in the mesHMLE-QKI-KO-iQKI-6 and -iQKI-7 cells (see Supplemental Figure 3). The resulting mesHMLE-QKI-KO-cQKI-5-iQKI-6 and mesHMLE-KO-KO-cQKI-5-iQKI-7 cell lines were cultured with or without doxycycline and stained with a QKI-5-specific antibody and an antibody specific for the induced isoform. In these cell lines, QKI-5 is variably expressed, allowing us to compare the nuclear localization of QKI-6 or QKI-7 between cells with low or high QKI-5 expression. Although most cells expressed QKI-6 and QKI-7 in the cytoplasm, a subset of cells that had the brightest QKI-5 fluorescence appeared to express QKI-6 and QKI-7 in the nucleus after doxycycline treatment (Figure 5, A, B, D and E). To quantitate this observation, we divided individual cells into interquartile ranges of QKI-5 fluorescence intensity. Cells within the fourth quartile group with the highest QKI-5 expression had a significantly higher mean nuclear percentage of QKI-6 or QKI-7 (Figure 5, C and F), consistent with QKI-5 enhancing their nuclear accumulation.

### QKI isoforms cooperate to regulate mesenchymal cell migration

**FIGURE 6:**
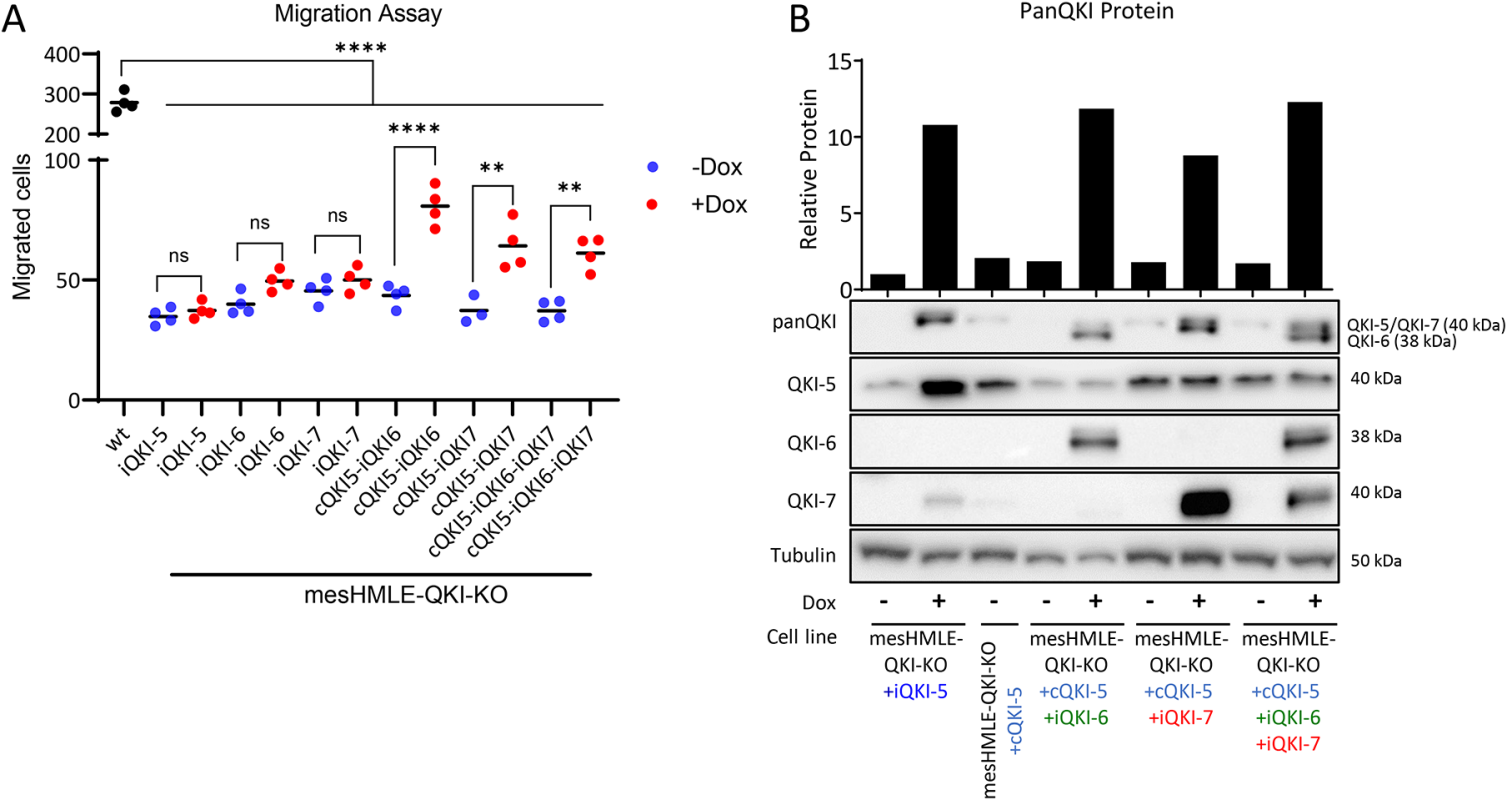
Coexpression of QKI isoforms collectively enhance mesenchymal cell migration. (A) Migration assay comparing wild type mesHMLE cells with derivatives of mesHMLE-QKI- treated with or without 1.00 µg/ml doxycycline, and with coexpression of constitutive QKI-5. Data are plotted as mean migrated cells per field. All experiments were performed with at least three replicates counting migrated cells in at least 10 fields per treatment. *P* values are calculated using one-way ANOVA (with Bonferroni multiple testing) and indicated as ns- not significant, **p < 0.01, and ****p < 0.0001. (B) Western blots showing QKI levels following expression of different combination of QKI isoforms in mesHMLE-QKI-KO cells used in migration assays. The panQKI western blot is quantitated in the upper panel.

The increased ability of cells to migrate is a hallmark of the mesenchymal phenotype. Knockdown of QKI has been shown to decrease the migratory capacity of mesenchymal cells (Pillman *et al.*, 2018), however little is known about the contribution of individual QKI isoforms to this phenotype. To determine whether the stable loss of QKI protein in the mesHMLE-QKI-KO cells could suppress normal levels of migration and if overexpression of QKI isoforms could rescue migration, wild-type mesHMLE or mesHMLE-QKI-KO cells with inducible expression of individual isoforms were plated in Transwell migration chambers and a 4-h migration assay was performed. Cell migration was dramatically reduced in all derivatives of mesHMLE-QKI-KO cells compared with wild-type mesHMLE cells (Figure 6A). Reexpression of individual QKI isoforms were unable to rescue this deficit in cell migration (Figure 6A), suggesting multiple QKI isoforms may coordinate this function in a normal cellular context. To test this concept, the migration assay was performed with cells in which two or three QKI isoforms were expressed. Western blot and qRT-PCR confirmed that each expected isoform was induced in these cell lines with total QKI levels being similar between each group (Figure 6B; Supplemental Figure 5). The expression of combinations of QKI isoforms was able to rescue migration to a greater degree than the individual isoforms (Figure 6A), although this was still significantly lower than the migration of wild-type cells most likely due to the variability in QKI isoform levels within individual cells (see Figure 5). These data are consistent with the QKI isoforms working cooperatively to promote migration in mesenchymal cells.

### QKI-5 plays a major role in maintaining mesenchymal cell morphology

**FIGURE 7:**
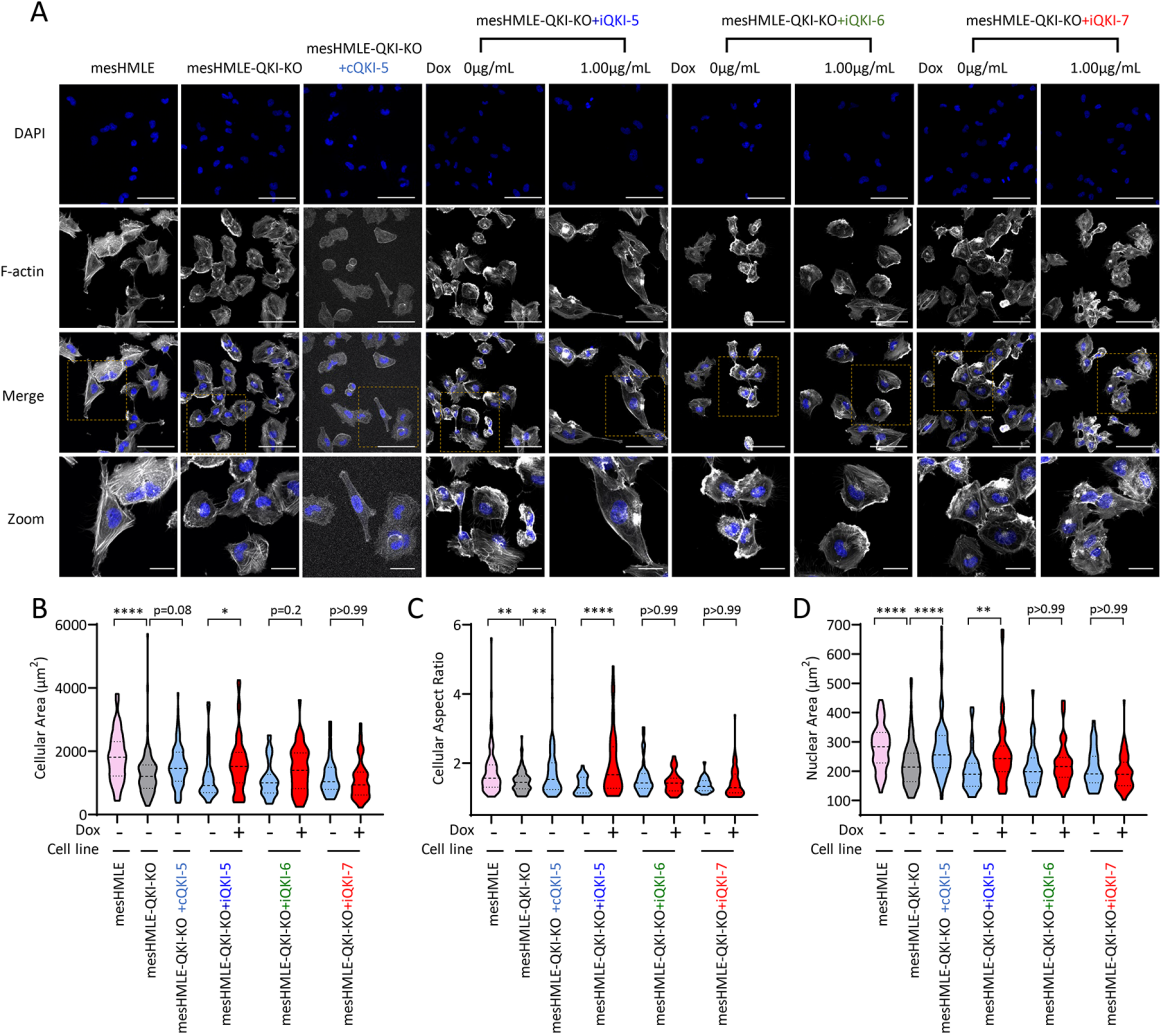
QKI-5 is required for mesenchymal morphology of mesHMLE cells. (A) Fluorescence microscopy of F-actin and nuclei (DAPI) in wild-type mesHMLE, mesHMLE-QKI-KO cells, and mesHMLE-QKI-KO cells with constitutive expression of QKI-5 or doxycycline induction of either QKI-5, QKI-6, or QKI-7. Merged images are a combination of F-actin and DAPI. Large scale bars represent 50 µm and small scale bars represent 5 µm. Violin plots show quantitated changes in (B) cellular area, (C) cellular aspect (length to width) ratio and (D) nuclear area, calculated on a minimum of 32 cells per condition. *P* values are calculated using one-way ANOVA (with Bonferroni multiple testing) and indicated as*p < 0.05, **p < 0.01, and ****p < 0.0001.

QKI-5 has been shown to be important for the maintenance of mesenchymal morphology, likely through direct regulation of alternative splicing of genes involved in the actin cytoskeletal pathway (Li *et al.*, 2018; Pillman *et al.*, 2018). However, a role for QKI-6 and QKI-7 in maintaining mesenchymal morphology has not been explored. To characterize the effect that loss of total QKI protein has on mesenchymal morphology, wild-type and mesHMLE-QKI-KO cells were stained for F-Actin and cell morphology analyzed. by measuring cellular area and length to width ratio (aspect ratio). QKI knockout cells exhibited statistically significant reductions in area and aspect ratio compared with wild-type cells, consistent with loss of QKI promoting epithelial morphology (Figure 7, A–C). Interestingly, the mesHMLE-QKI-KO cell nuclei displayed a significant reduction in area and an irregularly shaped morphology relative to wild-type cells (Figure 7D; Supplemental Figure 6). These data support an important role for QKI expression in maintaining mesenchymal cell morphology.

To determine whether reconstitution of QKI-5 can rescue the mesenchymal morphology lost in mesHMLE-QKI-KO cells, mesHMLE-QKI-KO-iQKI-5 cells were cultured with and without doxycycline and analyzed for changes in morphology. Reconstitution of QKI-5 caused a significant restoration of the mesenchymal morphology, marked by changes in cellular area and aspect ratio (Figure 7, A–C). In addition, there was a significant increase in nuclear area with inducible reconstitution of QKI-5 (Figure 7D; Supplemental Figure 6). Further supporting these findings, constitutive reconstitution of QKI-5 (mesHMLE-QKI-KO-cQKI-5) showed similar changes in nuclear and cell body morphology (Figure 7). Interestingly, expression of QKI-6 or QKI-7 did not cause an observable restoration of mesenchymal morphology and there were no significant changes in any of the morphological measurements (Figure 7B). Furthermore, neither QKI-6 nor QKI-7 expression caused an increase in nuclear area or an obvious rescue of nuclear morphology (Figure 7; Supplemental Figure 6). This suggests that QKI-5 expression is specifically required to rescue the changes in morphology seen in mesHMLE-QKI-KO cells.

**FIGURE 8:**
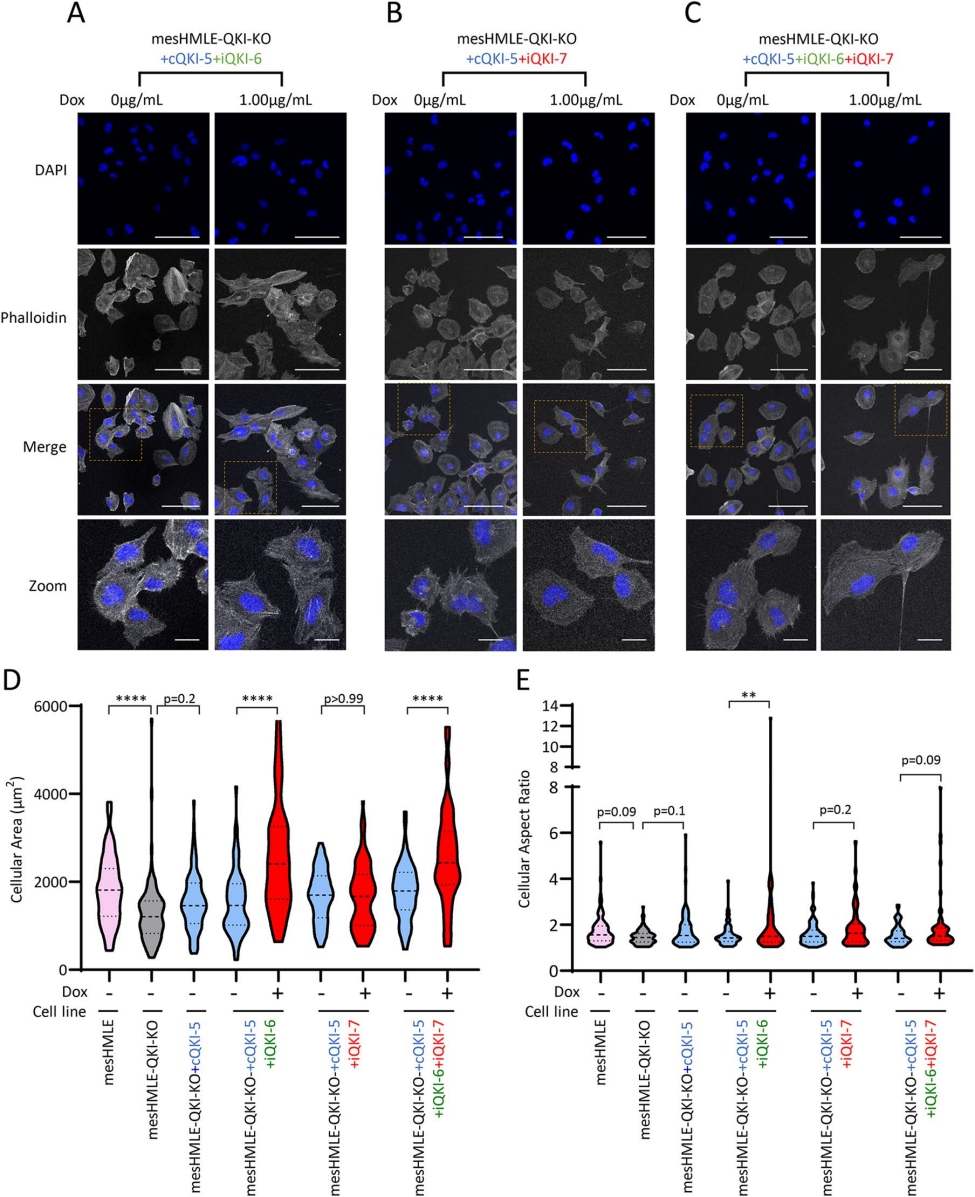
QKI-6 and -7 both affect mesenchymal morphology, when coexpressed with QKI-5. (A–C) Fluorescence microscopy of F-actin and nuclei (DAPI) in mesHMLE-QKI-KO cells with constitutive expression of QKI-5 and doxycycline inducible expression of QKI-6 and/or QKI-7. Merged images are a combination of F-actin and the stained QKI isoform. Large scale bars represent 50 µm and small scale bars represent 5 μm. Violin plots show quantitated changes in (D) cellular area and (E) cellular aspect ratio, calculated on a minimum of 32 cells per condition. For comparison, data for mesHMLE, mesHMLE-QKI-KO, and mesHMLE-QKI-KO-cQKI-5 are also shown (taken from Figure 7). *P* values are calculated using one-way ANOVA (with Bonferroni multiple testing) and indicated as **p < 0.01 and ****p < 0.0001.

The combined expression of QKI-6 or QKI-7 with QKI-5 causes a stronger rescue of the migration phenotype, suggesting that the QKI isoforms may cooperate to enhance the mesenchymal phenotype. To determine whether QKI-6 or QKI-7 could enhance mesenchymal morphology promoted by QKI-5 overexpression, cell morphology was examined after coexpression of the two combinations or all three QKI isoforms. Induction of QKI-6 along with constitutive QKI-5 expression increased cellular area and the cell length to width ratio to an extent greater than QKI-5 alone (Figure 8, A and E). In contrast, QKI-7 induction did not significantly enhance these features, suggesting QKI-6 may uniquely influence aspects of cell morphology (Figure 8, A and E). These results demonstrate that while QKI-5 is the major isoform impacting mesenchymal morphology, QKI-6 may enhance this function, indicative of a cooperative effect of the QKI isoforms in promoting mesenchymal cell morphology.

## DISCUSSION

EMT is a dynamic cellular transition contributing to disease progression and is regulated by widespread changes in gene transcription and alternative splicing (Neumann *et al.*, 2018; Stemmler *et al.*, 2019). Previous work has identified QKI-5 as an important regulator of mesenchymal cell alternative splicing (Yang *et al.*, 2016; Li *et al.*, 2018; Pillman *et al.*, 2018), however, the contribution of individual QKI isoforms to mesenchymal features has not been investigated. Here, we find that QKI expression is required to maintain the mesenchymal phenotype of mammary epithelial cells that have undergone a TGFβ-induced EMT, and that the cytoplasmic QKI-6 and QKI-7 cooperate with nuclear QKI-5 to enhance morphology and migration. These results suggests that the mechanisms through which QKI controls morphology and migration are not limited to alternative splicing, and potentially involve regulation of mRNA metabolism within the cytoplasm.

Intriguingly, although we found that QKI-5 causes the strongest effect on splicing, the cytoplasm-enriched isoforms, QKI-6 and QKI-7, can also participate in alternative splicing of the QKI targets ADD3 and NFYA (Yang *et al.*, 2016; Pillman *et al.*, 2018; Wang *et al.*, 2021). This finding is supported by previous work where QKI-6 was found to participate in splicing of HDAC7 in vascular cells (Caines *et al.*, 2019). Although it is possible that QKI-6 and QKI-7 may influence splicing indirectly we observed some nuclear staining of QKI-6 and QKI-7, which was increased in cells wither higher QKI-5 expression, indicative of a direct effect on splicing. Cytoplasmic-nuclear shuttling of QKI isoforms has been reported previously, where QKI-7 nuclear localization is dependent on QKI-5 in the NIH 3T3 fibroblast cell line and in primary rat oligodendrocytes (Pilotte *et al.*, 2001). It is possible that the overlap in function between the isoforms allows for the alternative splicing profile to be maintained in scenarios where there are higher levels of QKI-6 and QKI-7 and reduced levels of QKI-5.

While all three major QKI isoforms were able to participate in alternative splicing, QKI-5 was the only individual isoform able to rescue the loss of mesenchymal morphology seen in QKI knockout cells. As QKI-5 caused the greatest rescue of mesenchymal alternative splicing, one explanation is that the effect of QKI-5 on splicing reaches a threshold that is great enough to affect morphology, which QKI-6 and QKI-7 cannot meet. The enrichment of genes associated with the actin cytoskeleton within the splicing targets of QKI support the idea that QKI promotes mesenchymal morphology through alternative splicing (Pillman *et al.*, 2018).

One noteworthy observation is the change in nuclear size and morphology seen with loss of QKI in mesHMLE. A similar change in nuclear morphology was observed in HeLa cells after siRNA-mediated knockdown of PITPβ, which also caused a compaction of the Golgi apparatus and an effect on Golgi-endoplasmic reticulum retrograde trafficking of vesicles (Carvou *et al.*, 2010). Compaction of the Golgi apparatus has been reported to occur during EMT and to aid cancer cell migration and metastasis (Tan *et al.*, 2017), and it may be that QKI also has a role in regulating this phenomenon. The QKI splicing target MYO18A (Pillman *et al.*, 2018) has reported functions in the mechanics and shape of the Golgi but the function of its alternative isoforms are unknown in this context (Taft and Latham, 2020). Changes in nuclear size and morphology are also known to be influenced by alterations in cell shape and adhesion, with dynamic changes in the actin cytoskeleton coordinating these cellular features (Khatau *et al.*, 2009). Indeed, we observed cells with loss of QKI and reconstitution of QKI-5 displayed coordinated changes in both cellular and nuclear morphology and size. However, it is important to note that, although QKI expression is higher in mesenchymal cells, it is not absent from epithelial cells, and reducing the levels of QKI to lower than what is expressed in epithelial cells could cause consequences that are not relevant in a classical EMT.

The organization of the QKI locus coupled with intrinsic autoregulatory effects between the QKI isoforms leads to a complex relationship between transcription and gene product output. Higher expression of QKI being associated with an isoform switch, and change in localization, strongly suggests that QKI causes different effects on mRNA metabolism when its expression is high compared with scenarios when it is low. As QKI has reported functions in many contexts of cellular differentiation (Neumann *et al.*, 2022), it is possible that the dose-dependent switch in function reflects a need for different effects on mRNA metabolism in different stages of differentiation. QKI could allow complex posttranscriptional events that need to occur sequentially, and in a temporally constrained manner, to be driven from a single gene locus during differentiation. This highlights the importance of studying the functions of QKI using methods that consider its dynamic expression pattern and complex functions. In this regard, while we have attempted to study the effects of the QKI isoforms in isolation and in combination using an inducible overexpression system, the levels of QKI obtained were often supraphysiological and may not accurately recapitulate the dynamic patterns of expression observed in an endogenous setting.

While QKI-6 and QKI-7 influence EMT associated properties, it remains unclear what molecular mechanisms these isoforms operate through in this context. Previous studies of QKI-6 and QKI-7, in other contexts, have found these isoforms to regulate mRNA stability, localization, and translation (Neumann *et al.*, 2022). In the case of QKI-7, it has been reported to increase the lengths of poly(A) tails of its target mRNAs through a direct interaction with PAP-associated domain containing 4 (PAPD4; Yamagishi *et al.*, 2016). QKI-6 and -7 have also been implicated in controlling the nuclear export and myelin sheath-specific translation of Myelin basic protein (Mbp) mRNA in mouse oligodendrocytes (Larocque *et al.*, 2002). In contrast to morphology, rescue of migration in the QKI knockout cells appears to require coexpression of QKI-5 with either QKI-6 or QKI-7. This implicates a nonsplicing regulation of mRNA in promoting QKI-associated migration and suggests that both nuclear and cytoplasmic isoforms are required. It is possible that the QKI isoforms cooperate to control the local translation of mRNA to sites relevant to cell migration, like lamellipodia or cell-matrix adhesions, enhancing their activity or formation. Further research is required to determine the specific roles that QKI-6 and QKI-7 play in maintaining the mesenchymal phenotype.

## MATERIALS AND METHODS

### Cell culture

The mesenchymal epithelial cell line (mesHMLE) was derived by culturing immortalized human mammary epithelial cells (HMLE) in mesHMLE culturing media (DMEM/F12 supplemented with 5% fetal bovine serum (FBS), 20 ng/ml epidermal growth factor, 10 μg/ml insulin and 0.5 μg/ml hydrocortisone) with 2.5 ng/μl TGF-β1 for at least 14 d (Mani *et al.*, 2008). The mesHMLE-derived cell lines were cultured in mesHMLE culturing media without TGF-β1.

Human breast cancer cell lines were cultured as follows: T-47D, MDA-MB-361, MDA-MB-134, MCF7, ZR-75-1, MDA-MB-231, Hs578T, and MDA-MB-415 were maintained in DMEM supplemented with 10% FBS. CAL-51, CAL-120, MDA-MB-157, and MDA-MB-436 were maintained in Roswell Park Memorial Institute (RPMI) 1640 media supplemented with 10% FBS, 2 mM HEPES, and 0.3U insulin. BT-549 cells were maintained in RPMI supplemented with 10% FBS. SUM159PT cells were maintained in Ham's F12 supplemented with 5% FBS, 5 μg/ml insulin, and 1 μg/ml hydrocortisone. The human prostate cancer cell lines LNCaP and PC-3 cells were maintained in RPMI with 10% FBS. LNCaP-iQKI-5 cells were made as previously described (Pillman *et al.*, 2018). HEK293T cells were maintained in DMEM supplemented with 10% FBS.

### Generation of QKI knockout mesHMLE cell line with CRISPR-Cas9

The guide RNA sequence was designed to target the first exon of the QKI gene (Supplemental Table 1). The guide RNAs, tracrRNA and Cas9 protein were purchased from Integrated DNA Technologies (AltR system). The tracrRNA and guideRNA were combined and diluted to a 1 μM concentration in Nuclease-free Duplex buffer (IDT). These mixtures were then heated for 5 min at 95°C. Six microliter of the tracrRNA/guideRNA solution was combined with 6 μl of 1 μM Cas9 protein and then diluted with 88 μl of Opti-MEM Medium. The ribonucleoparticle (RNP) solution was incubated at room temperature for 5 min. MesHMLE cells (2 × 10^4^ cells) were plated in 24-wells at with 400 μl medium and transfected with the RNP solution using the RNAiMAX Transfection Reagent (Invitrogen). Following 72 h of culture, single clones were expanded and screened for loss of QKI protein by Western Blot. The genomic sequence spanning the predicted gRNA cut site of the selected clone was amplified by PCR and Sanger sequenced to assess CRISPR induced genomic changes. Primer sequences are in Supplemental Table 1.

### Cloning of inducible QKI isoform constructs

The ORFs for QKI-5, -6 and -7 were amplified by RT-PCR and cloned into the *Bam*HI/*Not*I sites of pENTR2B entry vector (Invitrogen). The primers used for PCR amplification are shown in Supplemental Table 1. The Gateway LR Clonase II Enzyme Mix (Invitrogen) was used to recombine the pENTR2B-QKI-5, pENTR2B-QKI-6, and pENTR2B-QKI-7 vectors into the pInducer20 expression vector (Addgene #44012), and the pENTR2B-QKI-5 vector into the pLX301 vector (Addgene #25895). Constructs were verified by Sanger sequencing.

### Transfection of siRNAs

Transfections of 20 nM of each siRNA was performed using Lipofectamine RNAiMAX according to the manufacturer's protocol (Thermo Fisher Scientific). Transfection media was removed 24 h after transfection and cellular material was harvested 72 h after transfection. Sequences of siRNAs used in this study are shown in Supplemental Table 1.

### Generation of lentiviral particles and viral transduction of cell lines

To produce lentiviruses, HEK293T cells (2 × 10^6^ cells in a T25 flask) were transfected with 4 µg of transfer vector and 1 μg each of pLP1, pLP2, pLP/VSVG, and pTAT using Lipofectamine 2000 (12 µL, Invitrogen) in a total of 4 ml media. Following 72 h, the lentivirus-containing medium was harvested and purified by filtration through a 0.45 µm for use in transductions.

To transduce target cells, 3 ml of mesHMLE culturing medium was combined with 1 ml of virus-containing medium and 4 μg/ml polybrene and added to sparsely plated mesHMLE cells. Cells were passaged upon confluence and grown for 24 h before selection medium was added (Puromycin: 1 µg/ml, Neomycin: 500 μg/ml).

### RNA Isolation, cDNA synthesis, and qRT-PCR

RNA was isolated from target cells using the TRIzol reagent following the standard manufacturer's protocol (Thermo Fisher Scientific). Complementary DNA was synthesized from 1 μg of RNA using the QuantitTect RT kit (Qiagen). Quantitative PCRs were performed in triplicate using the QuantiTect SYBR Green reagent (Qiagen) on a Rotor-Gene 6000 series thermocycler (Qiagen). Quantitative PCR analysis was computed using the comparative quantitation feature in the Rotor-Gene software and qPCR assays were normalized to GAPDH expression unless otherwise stated. Oligonucleotides are shown in Supplemental Table 1.

### Protein lysate purification and western blotting

Protein extracts were obtained by lysis of cells in 1xRIPA buffer (Abcam) containing protease (cOmplete Mini, EDTA-free Protease inhibitor Cocktail, Roche) and phosphatase (PhosSTOP EASYpack, Roche) inhibitors. Twenty micrograms of lysate were fractionated on an Invitrogen Bolt Bis-Tris Plus gel. Protein was transferred for 90 min at 4 °C at 250 mA on a magnetic stirrer. Membrane was blocked in 5% skim milk for 1 h at room temperature and probing was carried out at 4 °C overnight using the antibody dilutions described in Supplemental Table 2.

The enhanced chemiluminescence (ECL) method (Pierce) was used to visualize bands on the membranes, which were exposed on a ChemiDoc imaging system (Bio-Rad). Target protein bands were quantitated to α-Tubulin using FIJI software (Schindelin *et al.*, 2012).

### Immunofluorescence and microscopy

Cells were plated onto fibronectin-coated chamber slides (Nunc) and fixed with 4% paraformaldehyde after 24 h. Chambers were permeabilized with 0.1% Triton X-100 and probed with antibodies or phalloidin according to the concentrations in Supplemental Table 2. Primary antibodies were added to chambers and incubated for 1 h at room temperature and then detected following 1 h incubation with Alexa-fluor antibodies (Thermo Fisher Scientific). Slides were mounted to coverslips using ProLong Gold Antifade Reagent containing DAPI (Molecular Probes). Images were acquired using an LSM-700 confocal microscope (Zeiss).

### Image analysis

Fluorescence quantitation was performed using the FIJI (ImageJ) software (Schindelin *et al.*, 2012). Two methods were used to quantitate total and nuclear fluorescence per cell. For the first method, normalized fluorescent intensities were quantitated by determining total fluorescence per field and dividing by the number of nuclei captured in the field. This was performed on images derived from samples that were not stained with a marker capable of delineating cell boundaries (Figure 4). Nuclear fluorescence was determined by generating a nuclear mask from the DAPI staining. Nuclear fluorescence per cell was calculated by determining total fluorescence contained within the mask and normalizing to the number of stained nuclei captured in the field.

Alternatively, total and nuclear fluorescence per cell was quantitated on a single-cell basis by manually tracing the boundary of each cell within a field and generating a nuclear mask from DAPI staining. Total fluorescence for a given cell was determined from the fluorescent intensity contained within a traced cell, while nuclear fluorescence was determined from the intensities contained within the nuclear mask and cytoplasmic fluorescence was calculated by subtracting the nuclear from total fluorescence.

To assess cell morphology, the cell area, nuclear area, and aspect ratio were calculated from the nuclear masks and cell traces using ImageJ.

### Cell migration assays

Cells were plated in 6.5 mm Transwells with 8.0-μm pores (Corning) with serum-free medium. Serum-containing medium (10% fetal calf serum) was added to the wells beneath each chamber to induce chemotactic migration across the Transwell membrane and after 4 h the Transwells were fixed by washing in 10% buffered formalin. Transwell membranes were excised with a scalpel, permeabilized with 0.1% Triton X in 1 × phosphate-buffered saline and mounted to slides and coverslips using ProLong Gold Antifade Reagent containing DAPI (Molecular Probes). Cells were imaged on an LSM-700 confocal microscope and cells were counted using FIJI software. All experiments included either three or four replicates.

### Statistical analysis

Patient RNA-Seq data from the Cancer Cell Line Encyclopedia (Barretina *et al.*, 2012) and the GTEx project (Consortium, 2013) were analyzed using the programming language Python with the modules Pandas (McKinney, 2010) and Numpy (Harris *et al.*, 2020). Statistics were performed using SciPy and Statannot. Charts were plotted using Seaborn and GraphPad Prism.

## Supplementary Material





## Abbreviations used:

CCLE: Cancer Cell Line Encyclopedia
CRISPR: clustered regularly interspaced short palindromic repeats
EMT: epithelial-mesenchymal transition
gRNA: guide RNA
GTEx: Genotype-Tissue Expression
KO: knockout
mesHMLE: mesenchymal human mammary epithelial cells
