# Supplementary material for: Quaking isoforms cooperate to promote the mesenchymal phenotype
Source: Mol Biol Cell. 2024 Jan 12;35(2):ar17. doi: 10.1091/mbc.E23-08-0316 (PMC10881146; doi:10.1091/mbc.E23-08-0316)
Supplement: Supplementary file 1 [file mbc-35-ar17-s001.pdf]

Supplemental Materials

Molecular Biology of the Cell

Neumann *et al*.

Supplementary Figure Legends

Supplementary Figure 1: Expression of the QKI isoforms in human tissues and cancer cell lines

(A) Expression of QKI isoforms in a panel of epithelial and mesenchymal breast cancer cell lines. (B) Relative expression of QKI isoform mRNA, (C) QKI isoform mRNA as a percentage of total QKI transcripts and (D) total QKI mRNA shown for prostate, pancreas, bladder, skin, ovary, breast, muscle, lung, brain, and nerve tissue samples mRNA in the Genotype-Tissue Expression (GTEx) project displayed as box and violin plots. (E) Relative expression of QKI isoform mRNA, (F) QKI isoform as a percentage of total QKI transcripts and (G) total QKI mRNA in the Cancer Cell line Encyclopedia (CCLE) displayed as box and violin plots. Data is shown for prostate adenocarcinoma (PRAD), small cell lung cancer (SCLC), breast carcinoma (BRCA), lung adenocarcinoma (LUAD), uterine corpus endometrial carcinoma (UCEC), ovarian carcinoma (OV), bladder carcinoma (BLCA), skin cutaneous melanoma (SKCM), glioblastoma (GBM) and lower grade glioma (LGG) tumour samples displayed as box and violin plots.

Supplementary Figure 2 – Characterization of the allelic variation in the mesHMLE-QKI-KO cell line

(A) Sanger sequencing was performed on a PCR product generated using a forward primer beginning at the start codon of the QKI open reading frame (ORF) and a reverse primer designed at 230 base pairs downstream. (B) Allele 1, 2 and 3 indicating specific sequence alterations are shown. Wild-type sequence and QKI gRNA (including PAM site, underlined) are indicated for comparison. The theoretical cut site is shown with a blue arrow on the QKI gRNA sequence. (C) Allele 1, 2 and 3 nucleotide sequence and their translated amino acid sequence compared with wild-type sequence.

Supplementary Figure 3 – Generation of QKI isoform specific overexpression cell lines

Schematic of the doxycycline inducible and constitutive QKI isoform overexpression vectors are shown. Each QKI isoform specific cell line was generated in the mesHMLE QKI-KO (clone #2) background with the combinations of vectors contained within each cell line indicated.

Supplementary Figure 4 – Dose dependent expression of each QKI isoform and their impact on ADD3 and NFYA alternative splicing

Bar plots depicting expression of QKI isoforms and ADD3 and NFYA splice events in wild-type mesHMLE cells and mesHMLE-QKI-KO-iQKI-5, -iQKI-6 or iQKI-7 cells cultured for 72 hours

without or with 0.05, 0.20 or 1.00 µg/mL doxycycline. Each column represents data from overexpression of a specific QKI isoform (shown in panels F, L, and R). The alternative splicing data are presented as percent spliced in (PSI) and on a per isoform basis for ADD3 (panels A, B, G, H, M and N) and NFYA (panels C, D, I, J, O and P). The relative impact of expression of one QKI isoform on the other QKI isoforms are shown in panels E, K, and Q. Data for qRT-PCRs is represented as the mean of three technical replicates -/+ SD.

Supplementary Figure 5: Quantitation of QKI isoforms levels following their combination expression in QKI knockout cells

QuantitativeRT-PCRs showing QKI levels following expression of different combination of QKI isoforms in mesHMLE-QKI-KO cells used in migration assays (Figure 6). All treatments were with 1.00 µg/mL doxycycline for 72 hours. Data for qRT-PCRs is represented as the mean of three technical replicates -/+ SD.

Supplementary Figure 6 – Changes in nuclear morphology and size in QKI knockout cells and after reconstitution of individual QKI isoforms

Fluorescence microscopy of DAPI staining in mesHMLE wild-type, QKI knockout cells, and QKI knockout cells with constitutive or inducible reconstitution of QKI isoforms. All treatments were with 1.00 µg/mL doxycycline for 72 hours. Large scale bars represent 50 µm and small scale bars represent 5 µm.

Supplementary Figure 1

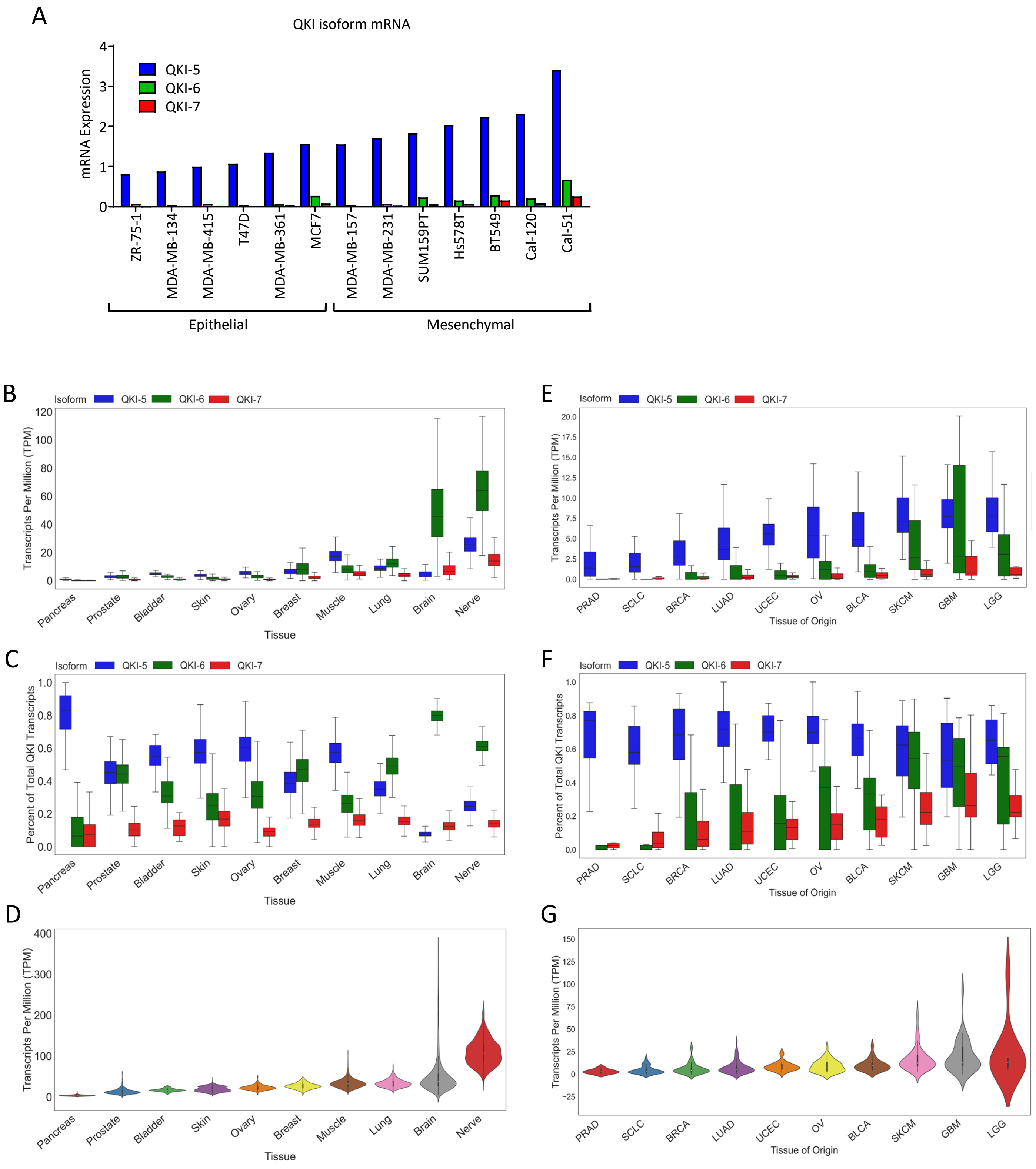

Supplementary Figure 2

A

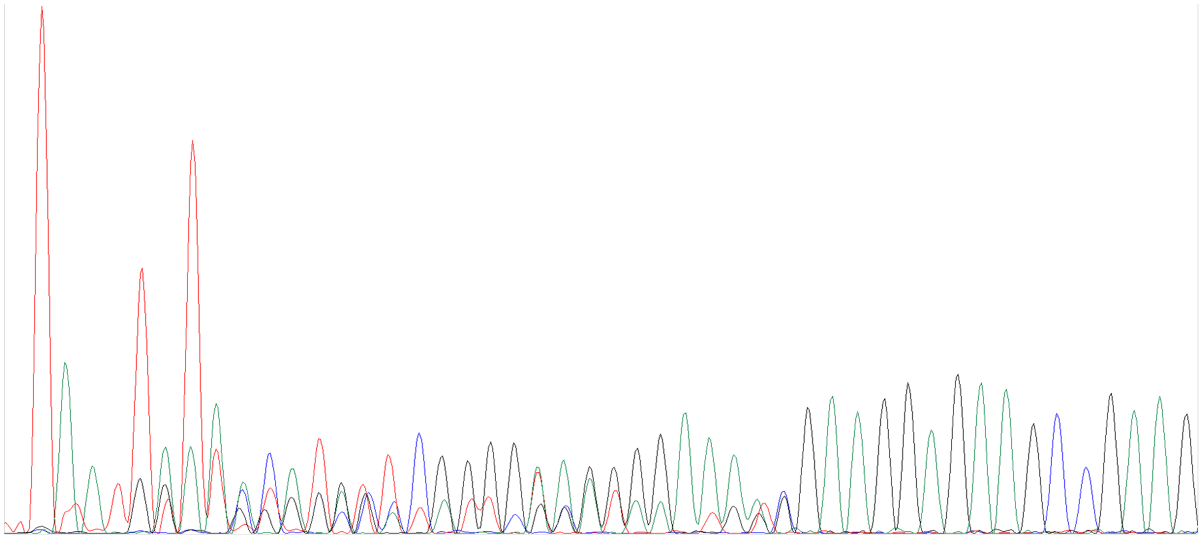

Allele 1 N N N N N N N N N N N N N N N N A T G G T C G G G G A A A T G G A A G G A G A A G C C G A A G
Allele 2 N N N N N N N N N N N N N N A T G G T C G G G G A A A T G G C G A A G G A G A A G C C G A A G
Allele 3 N N N N N N N N N N A T G G T C G G G G A A A T G G A A A A C G A A G G A G A A G C C G A A G

B

Nucleotide Sequence

guideRNA G GTC GGG GAA ATG GAA ACG A<u>AG G</u>

WT ATG GTC GGG GAA ATG GAA ACG AAG GAG AAG CCG AAG

Allele 1 ATG GTC GGG GAA ATG G AAG GAG AAG CCG AAG 5 Nucleotide Deletion

Allele 2 ATG GTC GGG GAA ATG G CG AAG GAG AAG CCG AAG 3 Nucleotide Deletion

Allele 3 ATG GTC GGG GAA ATG GAA^{A}ACG AAG GAG AAG CCG AAG 1 Nucleotide Insertion

C

Amino Acid Sequence

M V G E M E T K E K P K

WT ATG GTC GGG GAA ATG GAA ACG AAG GAG AAG CCG AAG

M V G E M E G E A E

Allele 1 ATG GTC GGG GAA ATG GAA GGA GAA GCC GAA G 5 Nucleotide Deletion

M V G E M A K E K P K

Allele 2 ATG GTC GGG GAA ATG GCG AAG GAG AAG CCG AAG 3 Nucleotide Deletion

M V G E M E N E G E A E

Allele 3 ATG GTC GGG GAA ATG GAA AAC GAA GGA GAA GCC GAA G 1 Nucleotide Insertion

Supplementary Figure 3

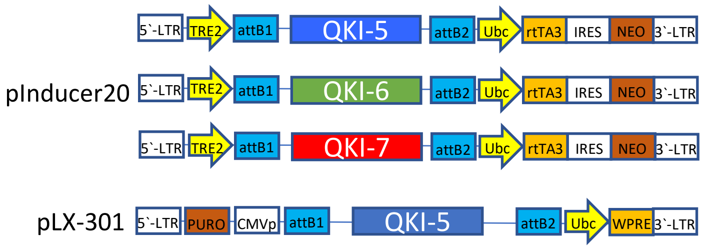

Cell lines generated in mesHMLE-QKI-KO background

	iQKI-5	+iQKI-6	+iQKI-7	cQKI-5	cQKI-5 +iQKI-6	cQKI-5 +iQKI-7	cQKI-5 +iQKI-6 +iQKI-7
pInducer20 QKI-5	+						
pInducer20 QKI-6		+			+		+
pInducer20 QKI-7			+			+	+
pLX-301 QKI-5				+	+	+	+

Supplementary Figure 4

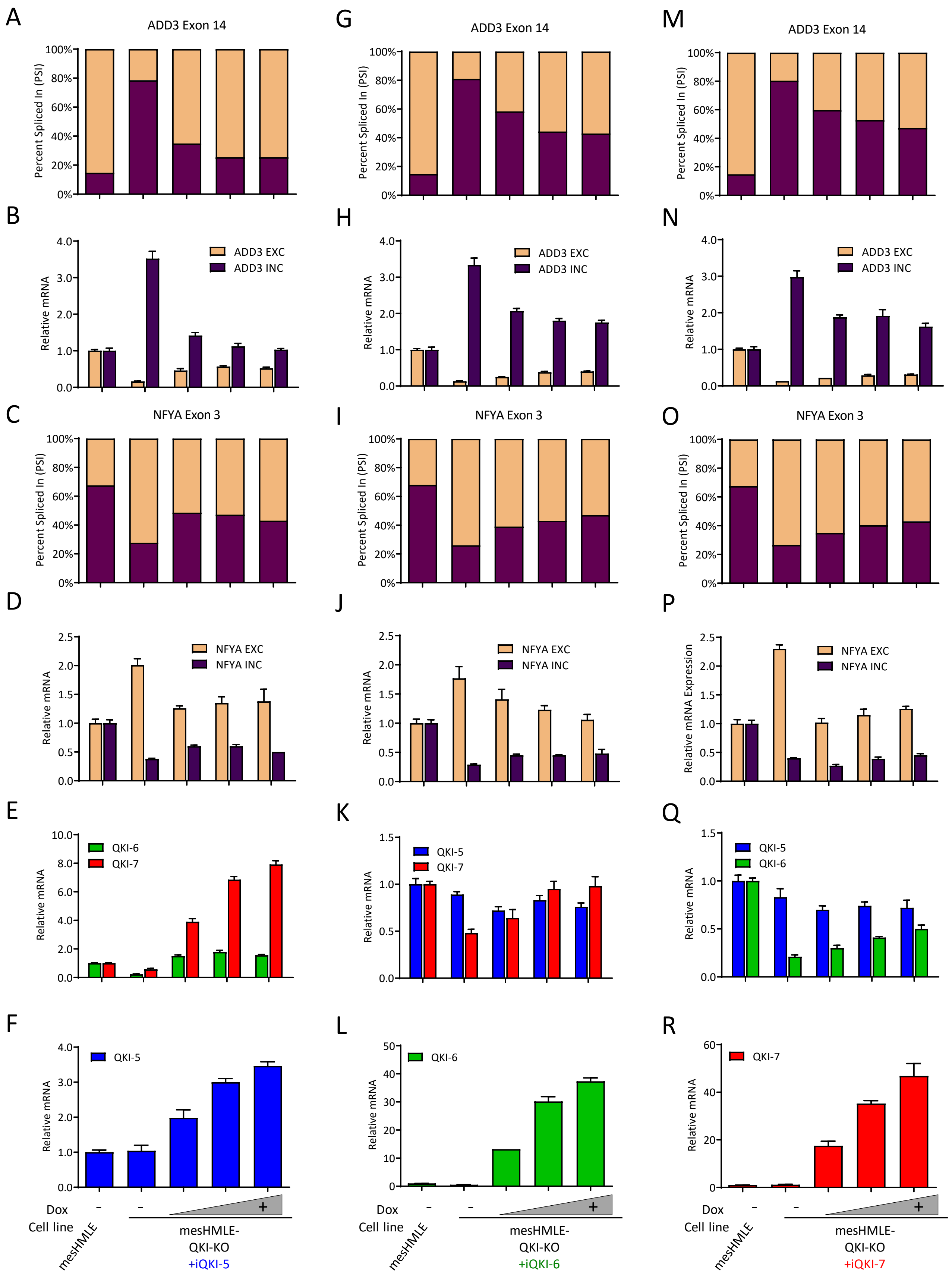

Supplementary Figure 5

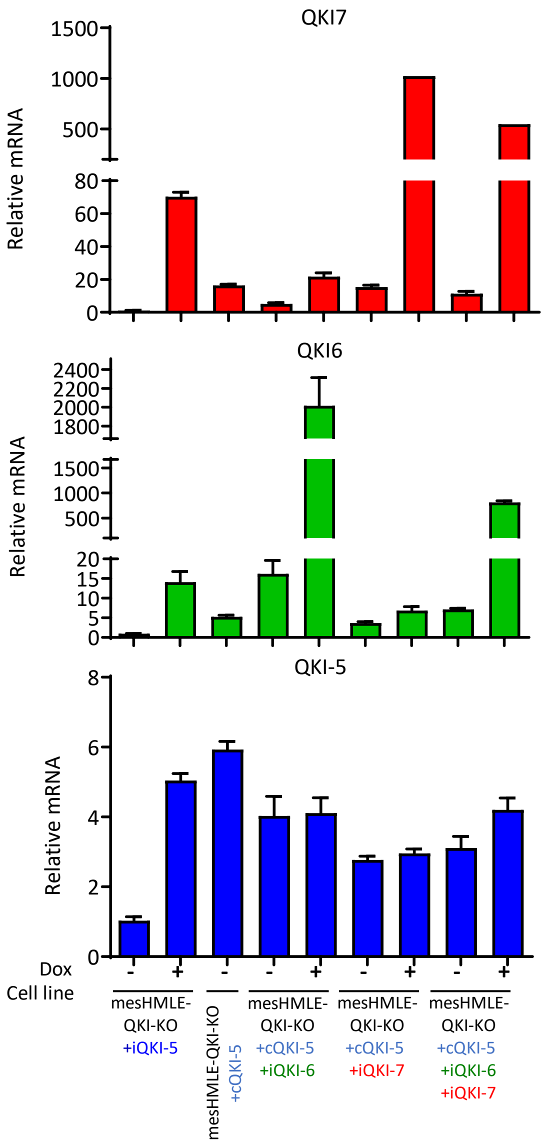

Supplementary Figure 6

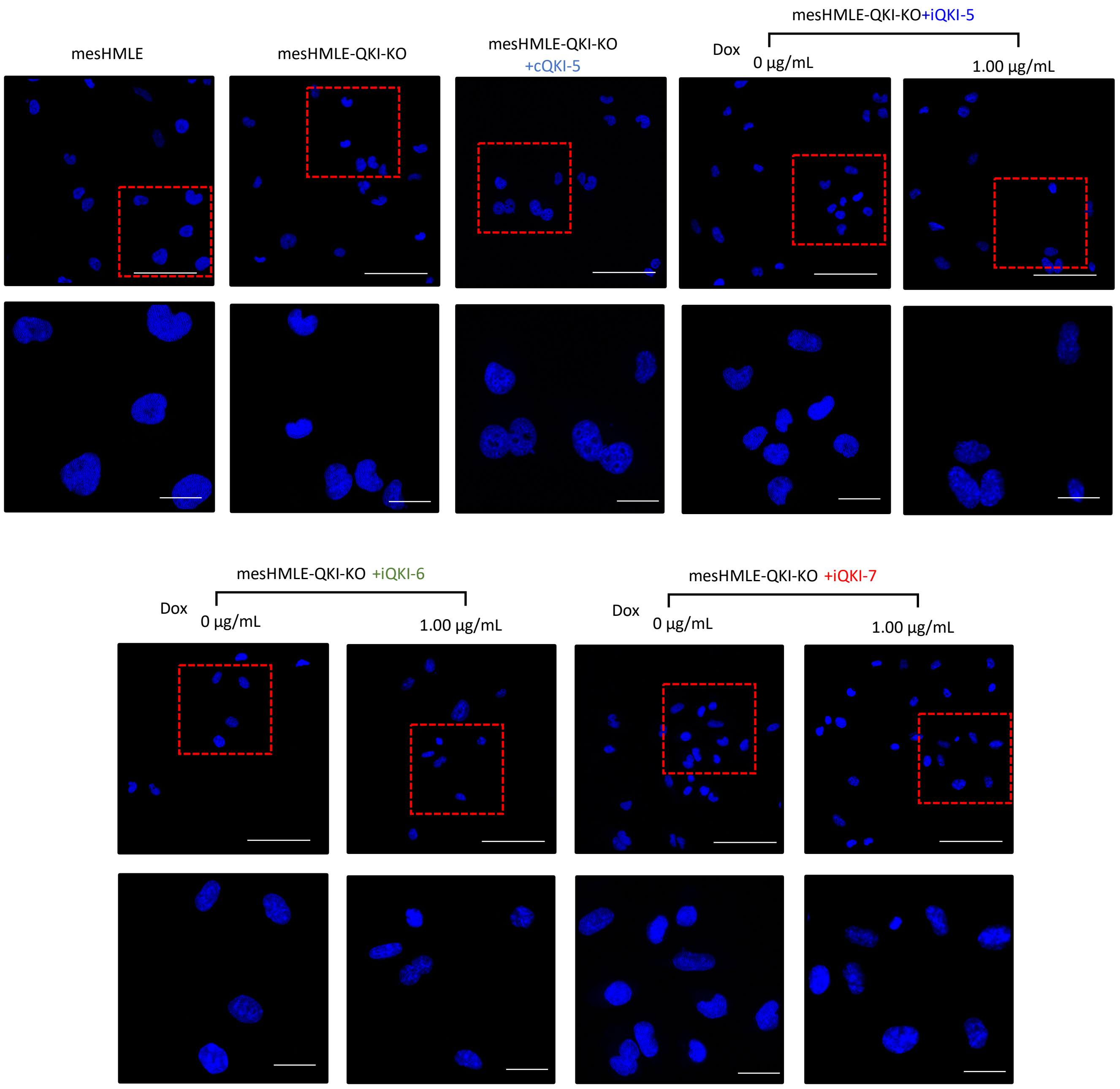

Supplementary Table 1: List of Oligonucleotides

APPLICATION	TARGET GENE	PRIMER NAME	SEQUENCE (5'-3')
qPCR	pan-Quaking (panQKI)	hqQKI F	CCTTGCCTTTTCTCTTGCAG
		hqQKI R	TATTGCAGCAGTTGGGTGAG
qPCR	Quaking-5 (QKI-5)	hqQKI-5 F	AATCCTTGAGTATCCTATTGAACCTAGT
		hqQKI-5 R	GCATATCGTGCCTTCGAACTTT
qPCR	Quaking-6 (QKI-6)	hqQKI-6 F	AGCTACATCAATCCTTGAGTATCCTATTG
		hqQKI-6 R	TAGCCTTTCGTTGGGAAAGC
qPCR	Quaking-7 (QKI-7)	hqQKI-7 F	GCTACATCAATCCTTGAGTATCCTATTG
		hqQKI-7 R	CAGGCATGACTGGCATTTCA
qPCR	Quaking-5 (QKI-5) endogenous mRNA	hqQKI-5_endo_F	CAGACCGAGCCGCCAC
		hqQKI-5_endo_R	GCAAGCAAAGGCGATTACCA
qPCR	Glyceraldehyde 3-phosphate dehydrogenase (GAPDH) (normaliser)	hqGAPDH F	ACCCAGAAGACTGTGGATGG
		hqGAPDH R	CAGTGAGCTTCCCGTTCAG
qPCR	Gamma Adducin (ADD3) exon 14 inclusion	hqADD3 ex13-14 F	ACCAGCTCCTCCTAACCCAT
		hqADD3 ex13-14 R	TCAGGGACATTTTGCGGTGA
qPCR	Gamma Adducin (ADD3) exon 14 exclusion	hqADD3 ex13-15 F	ACAACAAGGCCTAGAAGAAAACCA
		hqADD3 ex13-15 R	GTTGTACTTGTGCTTAACCTACTCA
qPCR	Nuclear Transcription Factor Y Subunit Alpha (NFYA) exon 3 inclusion	hqNFYA ex2-3 F	ACAGAGCAGATTGTTGTCCAGG
		hqNFYA ex2-3 R	CTACCTGGAGGGTCTGGACTT
qPCR	Nuclear Transcription Factor Y Subunit Alpha (NFYA) exon 3 exclusion	hqNFYA ex2-4 F	GATTCAGCAGCAGGTCCAAG
		hqNFYA ex2-4 R	TGCATGATGGTTTGACCTTGTC
CRISPR	Quaking guideRNA	QKI gRNA	GGTCGGGGAAATGGAAACGA
Sanger sequencing	Quaking (QKI)	QKI_KO_verificaion_rev	ATCAGGCAATTCTGCACTCC
APPLICATION	TARGET GENE	NAME	SUPPLIER, CAT #, SEQUENCE (5'-3')
siRNA	Non-targeting	Neg siRNA #1	Dharmacon, D-001810-10
siRNA	Non-targeting	Neg siRNA #2	GenePharma, UUCUCCGAACGUGUCACGU TT
siRNA	Quaking-5 (QKI-5)	siQKI-5 #1	Dharmacon, CUAUUAACCCACAGCAUUA UU
siRNA	Quaking-5 (QKI-5)	siQKI-5 #2	Dharmacon, CUGGUAAUCGCCUUUGCUU UU
siRNA	pan-Quaking (panQKI)	siQKIpan	Dharmacon, L-024905-01 (Smart Pool)
APPLICATION	TARGET GENE	NAME	SEQUENCE (5'-3')
Cloning	Quaking-5 (QKI-5)	hQKIorf F	AATGGATCCATGGTCGGGGAAATGGAA
		hQKI-5orf R	ATGCGGCCGCTTAGTTGCCGGTGGCGG
Cloning	Quaking-6 (QKI-6)	hQKIorf F	AATGGATCCATGGTCGGGGAAATGGAA
		hQKI-6orf R	ATGCGGCCGCTTAGCCTTTCGTTGGGAA
Cloning	Quaking-7 (QKI-7)	hQKIorf F	AATGGATCCATGGTCGGGGAAATGGAA
		hQKI-7orf R	ATGCGGCCGCTCAATGGGCTGAAATATC

Supplementary Table 2: Primary and secondary antibodies

<u>Western Blot</u>

**Target Protein**	**Animal**	**Brand**	**Dilution**	**Solution**	**Time**	**Temp**
α-Tubulin	Mouse	Abcam	1:5000	5%SMP/TNT	1hr	RT
α-panQKI	Mouse	Neuromab	1:5000	5%SMP/TNT	Overnight	4 °C
α-QKI-5	Mouse	Neuromab	1:5000	5%SMP/TNT	Overnight	4 °C
α-QKI-6	Mouse	Neuromab	1:1000	5%SMP/TNT	Overnight	4 °C
α-QKI-7	Mouse	Neuromab	1:1000	5%SMP/TNT	Overnight	4 °C
**Secondary Ab**	**Animal**	***Brand***	***Dilution***	***Solution***	***Time***	***Temp***
α-mouse HRP	Goat	ThermoFisher	1:10000	5%SMP/TNT	1hr	RT
α-Rabbit HRP	Goat	ThermoFisher	1:10000	5%SMP/TNT	1hr	RT

<u>Immunoflourescence</u>

Antibody	Animal	Brand	*Dilution*	*Time*	*Temp*
α-QKI-5	Rabbit	Bethyl	1:500	1hr	RT
α-QKI-5	Mouse	Neuromab	1:500	1hr	RT
α-QKI-6	Mouse	Neuromab	1:500	1hr	RT
α-QKI-7	Mouse	Neuromab	1:500	1hr	RT
α-mouse 594nm	Goat	ThermoFisher	1:500	1hr	RT
α-rabbit 647nm	Goat	ThermoFisher	1:500	1hr	RT
α-rabbit 594nm	Goat	ThermoFisher	1:500	1hr	RT
Phalloidin 647	N/A	ThermoFisher	1:500	1hr	RT
