# Supplementary material for: Quaking isoforms cooperate to promote the mesenchymal phenotype
Source: Mol Biol Cell. 2024 Jan 12;35(2):ar17. doi: 10.1091/mbc.E23-08-0316 (PMC10881146; doi:10.1091/mbc.E23-08-0316)
Supplement: Supplementary file 2 [file mbc-35-ar17-s002.pdf]

Quaking isoforms cooperate to promote the mesenchymal phenotype

Daniel Neumann, Caroline Phillips, Rachael Lumb, Helen Palethorpe, Yesha Ramani, Brett Hollier, Luke Selth, Cameron Bracken, Gregory Goodall, and Philip Gregory

Corresponding author(s): Philip Gregory, University of South Australia

Review Timeline:

Submission Date:	2023-08-15
Editorial Decision:	2023-09-11
Revision Received:	2023-10-17
Accepted:	2023-11-17

Editor-in-Chief: Matthew Welch

Transaction Report:

(Note: With the exception of the correction of typographical or spelling errors that could be a source of ambiguity, letters and reports are not edited. The original formatting of letters and referee reports may not be reflected in this compilation.)

RE: Manuscript #E23-08-0316
TITLE: Quaking isoforms cooperate to promote the mesenchymal phenotype

Dear Dr. Gregory:

The reviewers have raised a number of important points that need to be addressed through addition of new data as appropriate. The question raised by Reviewer 2 inquiring whether the total amount of QKI or the combination of isoforms is critical for the conclusions drawn is important and may require additional experiments. Please also address organizational points and provide an overview of the structure as suggested by Reviewer 1. There are some inherent caveats of the approach that should be mentioned in the Discussion as pointed out by Reviewer 1. A revised submission will only be successful if the points raised are addressed in a meaningful manner.

Sincerely,

Anita Corbett
Monitoring Editor
Molecular Biology of the Cell

Dear Dr. Gregory,

The review of your manuscript, referenced above, is now complete. The Monitoring Editor has decided that your manuscript is not acceptable for publication at this time, but may be deemed acceptable after specific revisions are made, as described in the Monitoring Editor's decision letter above and the reviewer comments below.

A reminder: Please do not contact the Monitoring Editor directly regarding your manuscript. If you have any questions regarding the review process or the decision, please contact the MBoC Editorial Office (mboc@ascb.org).

When submitting your revision include a rebuttal letter that details, point-by-point, how the Monitoring Editor's and reviewers' comments have been addressed. (The file type for this letter must be "rebuttal letter"; do not include your response to the Monitoring Editor and reviewers in a "cover letter.") Please bear in mind that your rebuttal letter will be published with your paper if it is accepted, unless you have opted out of publishing the review history.

If your manuscript contains a Significance Statement, please make sure that it consists of three separate bullet points totaling a maximum of 100 words and addressing each of the following points (see https://www.molbiolcell.org/curation-tools):
First bullet: What is the background context? What gap in knowledge does this study addres?
Second bullet: What are the key findings? What is unique or new about the approach?
Third bullet: Why is this paper significant? How might it influence future research?

Authors are allowed 90 days to submit a revision. If this time period is inadequate, please contact us at mboc@ascb.org.

Revised manuscripts are assigned to the original Monitoring Editor whenever possible. However, special circumstances may preclude this. Also, revised manuscripts are often sent out for re-review, usually to the original reviewers when possible. The Monitoring Editor may solicit additional reviews if it is deemed necessary to render a completely informed decision.

In preparing your revised manuscript, please follow the instruction in the Information for Authors (www.molbiolcell.org/info-for-authors). In particular, to prepare for the possible acceptance of your revised manuscript, submit final, publication-quality figures with your revision as described.

To submit the rebuttal letter, revised manuscript, and figures, use this link: Link Not Available

MBoC offers the option to publish your work with immediate open access. Open access can increase the discoverability and usability of your research. If you would like to publish your paper with immediate open access but did not select this option during initial submission, please contact the editorial office at mbc@ascb.org.

Thank you for submitting your manuscript to MBoC. We look forward to receiving your revised paper.

Sincerely,

Eric Baker

Journal Production Manager
MBoC Editorial Office
mbc@ascb.org

Reviewer #1 (Remarks to the Author):

This study investigates the functions of individual QKI isoforms in EMT processes. Using QKI isoform-specific inducible cell lines Neumann et al. achieve a better understanding of the functions of QKI-5, -6 and -7. Main findings of their studies are that QKI5 is required to induce the mesenchymal morphology while combined expression of QKI-5 with -6 or -7 enhances mesenchymal morphology and cell migration. Interestingly, they also find that -6 and -7 partially localize to the nucleus and contribute to alternative splicing. The experiments are well-conducted and the results scientifically sound. The manuscript is well written.

My comments for further improvements are the following:
-It would be helpful to add a panel to figure 1 showing a graphical figure with the differences in the QKI5,6,7 structure (and also where the siRNAs bind).

- In Figure 2B, how come QKI-5, -6 and -7 are not reduced to the same extent? The gRNA targets an exon that is shared by all three isoforms.

- With respect to the experiments described in Fig 3 and 5, it would have been interesting to including a Qua1 domain mutant of QKI-5 to tackle the dimerization hypothesis of the QKI isoforms.

- A limitation of their study is that it is largely based on overexpression studies. Overexpression often reaches supra-physiological levels, and may lead to non-specific or even toxic effects. It is advised to add this limitation to their discussion section.

Reviewer #2 (Remarks to the Author):

The manuscript by Neumann et al addresses the role of specific quaking isoforms in mesenchymal phenotype. Based on previous studies revealing the mRNA splicing factor quaking (QKI) contributes specifically to mesenchymal phenotype, the investigators develop a clone using crispr cass technology to largely delete expression of all isoforms and then add individual isoforms (5, 6 and 7) back through inducible doxycycline expression vectors. They also included constitutive 5 expression with inducible 6 or 7. After first establishing expression patterns of isoforms and confirming isoform 5 controls splicing and expression of 6 and 7 as previously reported, the investigators explore the functional outcome of each individual isoform on location and the mesenchymal phenotype. The largest differences observed were localization of isoform 5 to nucleus whereas 6 and 7 were largely cytoplasmic unless high expression was induced or co-expression with 5 and then only in the high expressing cells. Isoform 6 may have had a specific effect on migration. Cell shape appeared largely controlled by isoform 5.

The study has a number of significant experimental design issues.

1) It is unclear in any of the studies using a combination of isoform 5 with 6 or 7 if the effect is due to some specific isoform combination or increasing the total amount of QKI. Indeed, the investigators show increasing dox induction of gene expression altering nuclear localization. Does cQKI5 plus iQKI5 have the same effect with dox addition on migration as cQKI5 plus iQKI6? Showing isoform specific response is the focus of the manuscript.
2) Fig 3B and C Investigators need to include dox control of parental cell line and QK1 KO to insure there is no dox effect.
3) Measures of gene markers of mesenchymal phenotype need to be added to figure 7 and 8.
4) What statistical test was used and did it incorporate the testing of multiple samples?
5) The title really does not reflect the data. No data revealed that the combination of cQKI5 and i6 or i7 affected the area or aspect ratio, in a statistically significant manner, over cQKI5 alone.

In addition, the following specific items need to be addressed:

1) Fig 3 panel A is not clearly labeled or organized. Please put protein data together and label all graphs. The Y axis varies so individual labeling is needed.
2) Figs 4 and 5, please indicate in figure legend how many cells were counted and if this was done in a masked fashion. What statistical test was used?
3) Fig 7 please replot as the differences are almost imperceptible on the y axis used.

RE: Manuscript #E23-08-0316
TITLE: Quaking isoforms cooperate to promote the mesenchymal phenotype

Dear Dr. Gregory:

The reviewers have raised a number of important points that need to be addressed through addition of new data as appropriate. The question raised by Reviewer 2 inquiring whether the total amount of QKI or the combination of isoforms is critical for the conclusions drawn is important and may require additional experiments. Please also address organizational points and provide an overview of the structure as suggested by Reviewer 1. There are some inherent caveats of the approach that should be mentioned in the Discussion as pointed out by Reviewer 1. A revised submission will only be successful if the points raised are addressed in a meaningful manner.

Sincerely,

Anita Corbett
Monitoring Editor
Molecular Biology of the Cell

Reviewer #1 (Remarks to the Author):

This study investigates the functions of individual QKI isoforms in EMT processes. Using QKI isoform-specific inducible cell lines Neumann et al. achieve a better understanding of the functions of QKI-5, -6 and -7. Main findings of their studies are that QKI5 is required to induce the mesenchymal morphology while combined expression of QKI-5 with -6 or -7 enhances mesenchymal morphology and cell migration. Interestingly, they also find that -6 and -7 partially localize to the nucleus and contribute to alternative splicing. The experiments are well-conducted and the results scientifically sound. The manuscript is well written.

My comments for further improvements are the following:
-It would be helpful to add a panel to figure 1 showing a graphical figure with the differences in the QKI5,6,7 structure (and also where the siRNAs bind).

- We have added a graphical figure to Figure 1 (panel A) of the QKI isoform gene structures and have included the locations of the gRNA, isoform specific primers and the QKI-5 siRNA sequences.

- In Figure 2B, how come QKI-5, -6 and -7 are not reduced to the same extent? The gRNA targets an exon that is shared by all three isoforms.

- It is not entirely clear why the QKI-5 mRNA is reduced to a lesser extent than QKI-6 and QKI-7 in the QKI ko clones compared to wild-type cells. The relative levels of QKI isoform mRNAs appear to correlate with the QKI protein levels within the QKI ko clones. QKI is known to self-splice its own pre-mRNA (as implicated in Figure 1) and perhaps the balance of the QKI protein and its pre-mRNA contributes to variable levels of the QKI mRNA isoforms within the different QKI ko clones.

- With respect to the experiments described in Fig 3 and 5, it would have been interesting to including a Qua1 domain mutant of QKI-5 to tackle the dimerization hypothesis of the QKI isoforms.

- This would be a good experiment to try although there are reports that QKI dimerisation mutants (eg. E48G) have reduced stability (Beuck et al., 2012 JMB) which may make interpretation of these experiments challenging.

  - A limitation of their study is that it is largely based on overexpression studies. Overexpression often reaches supra-physiological levels, and may lead to non-specific or even toxic effects. It is advised to add this limitation to their discussion section.

- We agree that this limitation should be included and have done so in the discussion as below. "……This highlights the importance of studying the functions of QKI using methods that consider its dynamic expression pattern and complex functions. In this regard, while we have attempted to study the effects of the QKI isoforms in isolation and in combination using an inducible overexpression system, the levels of QKI obtained were often supra-physiological and may not accurately recapitulate the dynamic patterns of expression observed in an endogenous setting".

Reviewer #2 (Remarks to the Author):

The manuscript by Neumann et al addresses the role of specific quaking isoforms in mesenchymal phenotype. Based on previous studies revealing the mRNA splicing factor quaking (QKI) contributes

specifically to mesenchymal phenotype, the investigators develop a clone using crispr cass technology to largely delete expression of all isoforms and then add individual isoforms (5, 6 and 7) back through inducible doxycycline expression vectors. They also included constitutive 5 expression with inducible 6 or 7. After first establishing expression patterns of isoforms and confirming isoform 5 controls splicing and expression of 6 and 7 as previously reported, the investigators explore the functional outcome of each individual isoform on location and the mesenchymal phenotype. The largest differences observed were localization of isoform 5 to nucleus whereas 6 and 7 were largely cytoplasmic unless high expression was induced or co-expression with 5 and then only in the high expressing cells. Isoform 6 may have had a specific effect on migration. Cell shape appeared largely controlled by isoform 5.

The study has a number of significant experimental design issues.

1) It is unclear in any of the studies using a combination of isoform 5 with 6 or 7 if the effect is due to some specific isoform combination or increasing the total amount of QKI. Indeed, the investigators show increasing dox induction of gene expression altering nuclear localization. Does cQKI5 plus iQKI5 have the same effect with dox addition on migration as cQKI5 plus iQKI6? Showing isoform specific response is the focus of the manuscript.

- We have now provided a new Western Blot (Figure 6B) showing that the total levels of QKI protein in the combination isoform treatments is very similar to the individual isoform treatments. This indicates that the effect of the different QKI isoform combinations on cell migration and morphology is not related to overall changes in QKI protein levels.

2) Fig 3B and C Investigators need to include dox control of parental cell line and QK1 KO to insure there is no dox effect.

- We have performed Dox treatment of parental and QKI KO lines (in a single biological replicate) and shown it does not impact QKI levels or splicing (see Reviewer Figure 1 below).

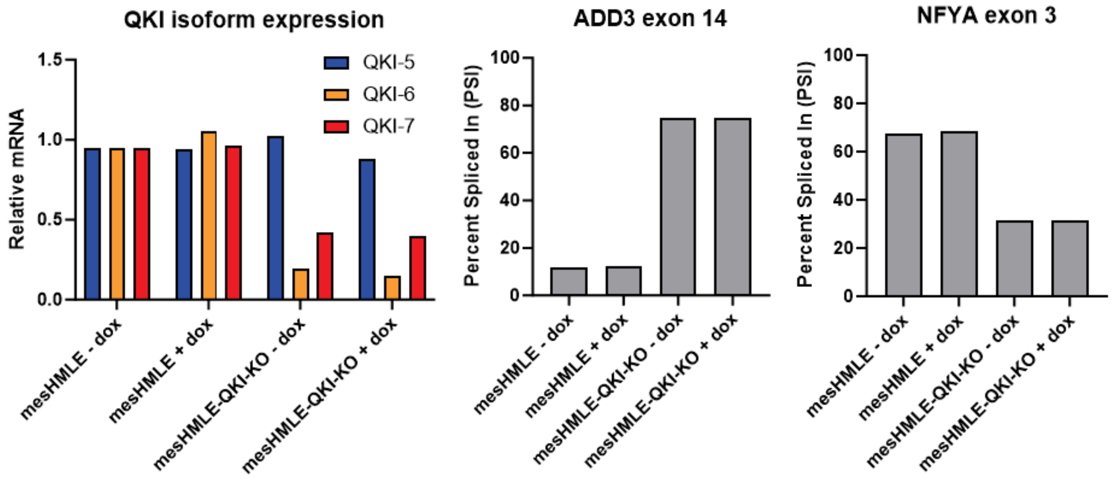

Reviewer Figure 1: QKI isoform and alternative spliced target expression in untreated or doxycycline treated cells. Wild-type mesHMLE or QKI knockout (ko) cells were treated with 1.00 µg/mL Dox for 72hr followed by qPCR of selected markers. Data represents the mean of one replicate.

3) Measures of gene markers of mesenchymal phenotype need to be added to figure 7 and 8.

- Mesenchymal marker gene expression is not influenced by QKI in mesHMLE and other mesenchymal breast cancer lines as shown in our previous work (Pillman et al., 2018 EMBO J). Instead, QKI influences mesenchymal morphology by regulating mesenchymal splicing patterns, as we have shown here in Figures 2, 3 and Supplementary Figure 4.

4) What statistical test was used and did it incorporate the testing of multiple samples?

- We have now included one way ANOVA analysis for all figures where multiple testing was applied including Bonferroni post-test for significance. These details have been added to the Figure Legends.

5) The title really does not reflect the data. No data revealed that the combination of cQKI5 and i6 or i7 affected the area or aspect ratio, in a statistically significant manner, over cQKI5 alone.

- The data in Figure 8 show induction of QKI-6 significantly influences cellular area and aspect ratio to a greater extent than cQKI-5 alone.

In addition, the following specific items need to be addressed:

1) Fig 3 panel A is not clearly labeled or organized. Please put protein data together and label all graphs. The Y axis varies so individual labeling is needed.

- We have re-organised Fig 3A so that all the protein data is together and all graphs are now clearly labeled.

2) Figs 4 and 5, please indicate in figure legend how many cells were counted and if this was done in a masked fashion. What statistical test was used?

- These details have now been added to the Figure Legends. Counting was not blinded but was semi-automated as described in the Methods.

3) Fig 7 please replot as the differences are almost imperceptible on the y axis used.

- We have re-plotted all of the violin plots in Figures 5, 7 and 8 on scales where differences between treatments are more visible.

RE: Manuscript #E23-08-0316R
TITLE: "Quaking isoforms cooperate to promote the mesenchymal phenotype"

Dear Dr. Gregory:

I am pleased to accept your manuscript for publication in Molecular Biology of the Cell.

Congratulations.

Sincerely,
Anita Corbett
Monitoring Editor
Molecular Biology of the Cell

Dear Dr. Gregory:

Congratulations on the acceptance of your manuscript! Thank you for choosing to publish your work in Molecular Biology of the Cell (MBoC).

Within 10 days, an unedited PDF of your manuscript will be published on MBoC in Press, an early release version of the journal. The date your manuscript appears on this site is the official publication date.

Your copyedited and typeset manuscript will be scheduled for publication in the next available issue of MBoC. Our production team will notify you when the page proofs of your paper are ready for your review.

MBoC offers the option to publish your paper with immediate open access. Open access can increase the discoverability and usability of your research. If you would like to publish your paper with immediate open access but did not select this option during initial submission, please contact the MBoC Editorial Office (mbc@ascb.org).

We invite you to submit images related to your accepted manuscript to be considered for the journal cover. Please contact mboc@ascb.org to learn how to submit candidate cover images.

Authors of Articles and Brief Communications are also encouraged to create a short video abstract to accompany their article when it is published. These video abstracts, known as Science Sketches, are up to 2 minutes long and will be published on YouTube and then embedded in the article abstract. Science Sketch Editors on the MBoC Editorial Board will provide guidance as you prepare your video. Information about how to prepare and submit a video abstract is available at www.molbiolcell.org/science-sketches. Please contact mboc@ascb.org if you are interested in creating a Science Sketch.

We look forward to publishing your paper in MBoC.
Sign up for MBoC's new content alerts here https://tinyurl.com/3bcsyk3e

Sincerely,

Eric Baker
Journal Production Manager
MBoC Editorial Office
mbc@ascb.org

Reviewer #1 (Remarks to the Author):

I have no further comments.
